# Targeting PI3K inhibitor resistance in breast cancer with metabolic drugs

**DOI:** 10.1038/s41392-025-02180-4

**Published:** 2025-03-21

**Authors:** Niklas Gremke, Isabelle Besong, Alina Stroh, Luise von Wichert, Marie Witt, Sabrina Elmshäuser, Michael Wanzel, Martin F. Fromm, R. Verena Taudte, Sabine Schmatloch, Thomas Karn, Mattea Reinisch, Nader Hirmas, Sibylle Loibl, Thomas Wündisch, Anne-Sophie Litmeyer, Paul Jank, Carsten Denkert, Sebastian Griewing, Uwe Wagner, Thorsten Stiewe

**Affiliations:** 1 Institute of Molecular Oncology, Universities of Gießen and Marburg Lung Center (UGMLC), Member of the German Center for Lung Research (DZL), Philipps-University, Marburg, Germany; 2 Department of Gynecology, Gynecological Endocrinology and Oncology, University Hospital Gießen and Marburg Campus Marburg, Philipps-University, Marburg, Germany; 3 Institute of Experimental and Clinical Pharmacology and Toxicology, Friedrich-Alexander-Universität Erlangen-Nürnberg, Erlangen, Germany; 4 FAU NeW – Research Center New Bioactive Compounds, Friedrich-Alexander-Universität Erlangen-Nürnberg, Erlangen, Germany; 5 Core Facility for Metabolomics, Philipps University, Marburg, Germany; 6 Elisabeth Hospital Kassel, Kassel, Germany; 7 UCT Frankfurt-Marburg, Department of Gynecology and Obstetrics, Goethe University, Frankfurt, Germany; 8 Breast Unit, University Hospital Mannheim, Mannheim, Germany; 9 Department of Gynecology with Breast Center, University Medicine Berlin, Berlin, Germany; 10 German Breast Group (GBG), Neu-Isenburg, Germany; 11 UCT Frankfurt-Marburg, Comprehensive Cancer Center Marburg, University Hospital Gießen and Marburg Campus Marburg, Philipps-University, Marburg, Germany; 12 Institute of Pathology, University Hospital Gießen and Marburg Campus Marburg, Philipps-University, Marburg, Germany; 13 Genomics Core Facility, Philipps-University, Marburg, Germany; 14 Institute of Lung Health, Justus Liebig University, Gießen, Germany

**Keywords:** Breast cancer, Target identification, Drug development

## Abstract

Activating *PIK3CA* mutations, present in up to 40% of hormone receptor-positive (HR^+^), human epidermal growth factor receptor 2-negative (Her2^−^) breast cancer (BC) patients, can be effectively targeted with the alpha isoform-specific PI3K inhibitor Alpelisib. This treatment significantly improves outcomes for HR^+^, Her2^−^, and *PIK3CA*-mutated metastatic BC patients. However, acquired resistance, often due to aberrant activation of the mTOR complex 1 (mTORC1) pathway, remains a significant clinical challenge. Our study, using in vitro and orthotopic xenograft mouse models, demonstrates that constitutively active mTORC1 signaling renders PI3K inhibitor-resistant BC exquisitely sensitive to various drugs targeting cancer metabolism. Mechanistically, mTORC1 suppresses the induction of autophagy during metabolic perturbation, leading to energy stress, a critical depletion of aspartate, and ultimately cell death. Supporting this mechanism, BC cells with CRISPR/Cas9-engineered knockouts of canonical autophagy genes showed similar vulnerability to metabolically active drugs. In BC patients, high mTORC1 activity, indicated by 4E-BP1^T37/46^ phosphorylation, correlated with p62 accumulation, a sign of impaired autophagy. Together, these markers predicted poor overall survival in multiple BC subgroups. Our findings reveal that aberrant mTORC1 signaling, a common cause of PI3K inhibitor resistance in BC, creates a druggable metabolic vulnerability by suppressing autophagy. Additionally, the combination of 4E-BP1^T37/46^ phosphorylation and p62 accumulation serves as a biomarker for poor overall survival, suggesting their potential utility in identifying BC patients who may benefit from metabolic therapies.

## Introduction

Breast cancer (BC) is the most commonly diagnosed cancer and the leading cause of cancer-related deaths among women globally.^1^ Based on hormone receptor (HR) and human epidermal growth factor receptor 2 (Her2) status BC is divided into four major molecular subtypes which dictate different clinical outcomes and guide patient’s choice of therapies: HR^+^/Her2^−^ (luminal A-like), HR^+^/Her2^+^ (luminal B-like), HR^−^/Her2^+^ (Her2-enriched), and HR^−^/Her2^−^ (triple-negative).^2,3^ More than 70% of all BC cases are HR^+^/Her2^−^ and up to 40% of these patients have activating mutations in the *PIK3CA* gene, which encodes the alpha isoform (p110α) of phosphatidylinositol 3-kinase (PI3K), crucial for cell proliferation, growth, survival, and metabolism.^4–6^ *PIK3CA* mutations (*PIK3CA*^mut^) most commonly occur as hotspot mutations in the catalytic domain of exon 20 (p.H1047R), followed by mutations in the helical domain of exon 9 (p.E542K and p.E545K) leading to activation of downstream signaling.^7^ In particular, *PIK3CA*^mut^ HR^+^/Her2^−^ metastatic BC (mBC) patients had a worse overall survival (OS) compared to *PIK3CA* wild-type patients (19.6 vs. 23.5 months, *p* = 0.04) and PI3K pathway activation predicted poor outcome after adjuvant endocrine therapy in HR^+^ BC patients.^8,9^ Hence, there is a strong rationale for therapeutically targeting the PI3K pathway, especially in HR^+^ BC, leading to the development of different PI3K inhibitors.^6^

The efficacy of the alpha isoform-specific PI3K inhibitor (PI3Ki) Alpelisib was evaluated in the phase 3 SOLAR-1 clinical trial where Alpelisib treatment in combination with Fulvestrant significantly prolonged progression-free survival (PFS) among patients with *PIK3CA*-mutated HR^+^/Her2^−^ mBC who had received endocrine therapy previously (11.0 vs. 5.7 months in the Placebo–Fulvestrant group, HR 0.65; 95% CI, 0.50 to 0.85; *p* < 0.001).^10,11^ However, acquired PI3Ki-resistance remains a significant clinical challenge, driven primarily by aberrant reactivation of the PI3K/AKT/mTOR pathway and activation of compensatory pro-survival mechanisms, including MAPK/MEK upregulation, JAK2/STAT5 signaling, and IL-6-STAT3 feedback.^12–14^

Physiologically, the mTOR (mechanistic target of rapamycin) kinase acts in two distinct complexes (mTORC1 and mTORC2) and is regulated by growth factors, nutrients, amino acids, and oxygen availability to guide cell metabolism and growth.^15,16^ Given the central role of mTORC1 as a rheostat of cell metabolism, mTORC1 activation by nutrients and growth factors promotes anabolic processes such as protein and lipid synthesis while inhibiting catabolic pathways, including the major cellular digestion process of macroautophagy (hereafter referred to as autophagy).^17^ During autophagy, double-membraned vesicles (autophagosomes) engulf cellular components and fuse with lysosomes, leading to the breakdown of their contents and the generation of metabolites to sustain cellular metabolism.^18^ Numerous autophagy-related genes (ATGs) and their core complexes orchestrate the autophagy cascade, playing crucial roles in its various stages of initiation (e.g., FIP200 in the ULK1 complex), nucleation (e.g., ATG14 in the PI3K complex), elongation (e.g., ATG7 in the LC3-conjugation system), fusion and degradation.^19^ Importantly, ULK1 (Unc-51 Like Autophagy Activating Kinase 1) serves as the key mediator of mTORC1 signaling to autophagy.^20^ As such, under nutrient-rich conditions, elevated mTORC1 activity inhibits autophagy by phosphorylating ULK1 at Ser757, thereby disrupting its interaction with AMP-activated protein kinase (AMPK).^21^ Conversely, in nutrient-poor conditions, AMPK activation fosters autophagy by directly phosphorylating ULK1 at Ser317 and inhibiting mTORC1.^22^

Given the prominent role of mTORC1 activation in Alpelisib-resistant *PIK3CA*^mut^ BC, preclinical studies have shown that combining Alpelisib with pharmacological mTORC1 blockade (RAD001) can reverse Alpelisib-resistance both in vitro and in vivo.^14^ However, the clinical success of novel dual PI3K/mTORC1 inhibitors like GDC-0980 (Apitolisib), PF-04691502 (Gedatolisib), and GSK2126458 (Omipalisib) is limited by more severe toxicities due to their broad effects, leading to treatment discontinuation or reduced dose intensity in many tumor patients.^23–25^ Therefore, we aimed to identify novel therapeutic options for therapy refractory BC patients with acquired mTORC1-dependent Alpelisib-resistance.

Considering the protective role of autophagy in metabolic homeostasis, we hypothesized that mTORC1-mediated autophagy inhibition would render Alpelisib-resistant BC cells vulnerable to metabolic perturbations.^26,27^ Mechanistically, autophagy affects multiple aspects of cancer metabolism. For example, autophagy limits glycolysis by selectively degrading hexokinase 2 (HK2), which catalyzes the first step in glycolysis, and 6-phosphofructo-2-kinase/fructose-2,6-bisphosphatase 3 (PFKFB3), which through production of fructose-2,6-bisphosphate activates phosphofructokinase-1 (PFK1), the rate-limiting enzyme in glycolysis.^18,28,29^ Furthermore, autophagy deficiency impairs macromolecule degradation, reducing substrate availability for the tricarboxylic acid (TCA) cycle, which in turn hinders electron transport chain (ETC) function and ATP production, thereby sensitizing cells to energy stress.^27,30^ These findings suggest that alterations in autophagy may sensitize cancer cells to compounds that target either glycolysis or mitochondrial respiration.

In general, metabolic reprogramming is one of the hallmarks of cancer and is characterized by the ability of tumors to produce vast amounts of biomass needed for maximum growth and proliferation.^31,32^ At the molecular level, this is achieved by an increased glycolytic rate to fuel branching pathways (e.g., pentose phosphate pathway) and altered anabolic TCA cycle metabolism, generating ATP, NADPH, and the building blocks used as precursors for macromolecule synthesis.^33^ These processes can be pharmacologically targeted with inhibitors of pyruvate dehydrogenase kinase, for example, dichloroacetate (DCA), promoting entry of the glycolytic end product pyruvate into the TCA cycle, or with biguanides like Metformin, which inhibit the mitochondrial ETC.^34^ While phase 2 clinical trials assessing the oncological efficacy of DCA are ongoing (NCT05120284), Metformin remains a frequently considered anti-cancer therapeutic due to its long-standing use as an antidiabetic drug with minimal side effects.^35^ Nonetheless, the optimal patient population for this metabolic targeting approach remains to be defined. In recent phase 2 studies, Metformin demonstrated anti-cancer efficacy by improving OS when added to standard-of-care treatment (SoC) lung and ovarian cancer patients.^36,37^ However, a randomized phase 3 placebo-controlled, double-blind trial revealed no significant improvement in invasive disease-free survival among high-risk operable breast cancer patients, importantly, without stratification based on specific molecular tumor characteristics.^35,38^ Collectively, these study results indicate that targeting metabolism necessitates a personalized medicine approach to identify molecular signaling alterations in cancer cells and tumor tissue that confer a druggable metabolic vulnerability, thereby creating a therapeutic window in molecular-defined subpopulations.^39^

Here, we demonstrate that PI3Ki-resistant BC shows increased sensitivity to various inhibitors of cancer metabolism due to mTORC1-driven autophagy inhibition. Highlighting an autophagy deficiency in BC as a metabolic sensitizing mechanism, CRISPR/Cas9-engineered knockout of essential canonical autophagy genes induced a strong metabolic liability, both in vitro and in vivo. Importantly, an analysis of over 1100 BC patients revealed a significant correlation between 4E-BP1^T37/46^ phosphorylation and p62 accumulation, both serving as markers for mTORC1 activity and autophagy deficiency, with high expression of these markers significantly predicting worse OS in BC patients. Together, these findings could help to identify patients who might benefit from metabolic drugs through a personalized approach targeting autophagy-deficient BC.

## Results

### Metabolic liability of Alpelisib-resistant breast cancer

**Fig. 1:**
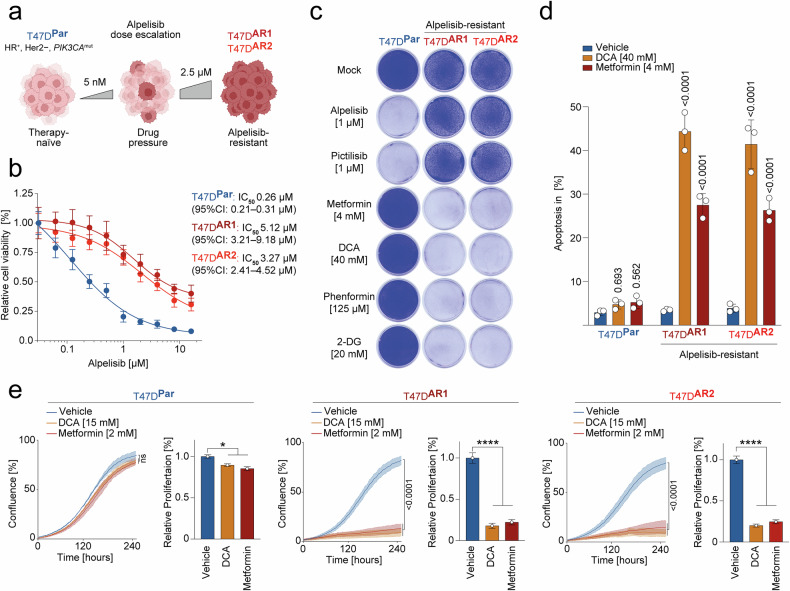
Metabolic vulnerability of Alpelisib-resistant breast cancer. **a** Schematic overview: Generation of Alpelisib-resistant breast cancer cells by dose escalation. Parental breast cancer cells (T47D^Par^) were treated with increasing doses of Alpelisib (5 nM to 2.5 µM) to obtain Alpelisib-resistant subclones (T47D^AR1^, T47D^AR2^). **b** Cell viability of parental and two Alpelisib-resistant subclones after 5-day treatment with Alpelisib. Shown are mean ± SD, *n* = 3, IC_50_ (95% CI). **c** Clonogenic growth of parental and Alpelisib-resistant cells treated with indicated drugs for 10 days. **d** Flow cytometry analysis for apoptosis (sub-G1). Indicated cells were treated with DCA or Metformin for 5 days. Shown are mean ± SD, *n* = 3, two-way ANOVA with Tukey’s multiple comparisons test. **e** Real-time live-cell imaging of parental (T47D^Par^) and Alpelisib-resistant cells (T47D^AR1^, T47D^AR2^) treated with DCA or Metformin. Shown is the mean confluence in % over time (*n* = 3) with FDR *q* values and the area under the proliferation curve (AUC) relative to untreated, one-way ANOVA with Dunnet’s multiple comparisons test, *p < 0.05; ****p < 0.0001

The worsened prognosis for BC patients with acquired targeted therapy resistance has prompted us to explore the vulnerabilities of PI3Ki-resistant breast cancer cells. T47D cells, derived from an HR^+^/Her2^−^ BC patient with a somatic *PIK3CA*^H1047R^ mutation, responded well to Alpelisib, a clinically approved molecular targeted drug for *PIK3CA*^mut^ HR^+^/Her2^−^ BC, with an IC_50_ of 0.26 µM (Fig. 1a, b).^40^ Using a dose escalation protocol, parental T47D cells (T47D^Par^) invariably adapted to Alpelisib, resulting in resistant subclones (T47D^AR1^ and T47D^AR2^) with more than 10-fold higher IC_50_ values compared to therapy-naïve T47D^Par^ cells (Fig. 1b).^14,41,42^ When examining the resistance pattern, T47D^AR1^ and T47D^AR2^ cells were not only resistant to the alpha isoform-specific PI3Ki Alpelisib but also cross-resistant to the pan-PI3Ki Pictilisib (Fig. 1c). In stark contrast, the clonogenic growth of T47D^AR1^ and T47D^AR2^ cells was severely inhibited by the biguanides Metformin and Phenformin, as well as the glycolysis inhibitors DCA and 2-deoxy-D-glucose (2-DG), despite minimal effects on therapy-naïve T47D^Par^ cells (Fig. 1c). In particular, the biguanides Metformin and Phenformin exert their anti-cancer effects through inhibition of mitochondrial ETC complex I whereby both 2-DG and DCA target glycolysis: 2-DG competes with hexokinase (HK), slowing glucose uptake, while DCA inhibits pyruvate dehydrogenase kinase (PDK), thereby reducing glycolytic lactate production and enhancing pyruvate oxidation.^35,43^

Detailed analysis of this specific metabolic liability revealed, that compared to T47D^Par^ cells, only Alpelisib-resistant cells showed significantly increased apoptosis and strongly diminished proliferation when treated with DCA or Metformin (Fig. 1d, e). Thus, employing varied analytical approaches, we concluded that Alpelisib-resistance engendered a marked vulnerability to perturbations in glucose metabolism and mitochondrial respiration.

### Metabolic vulnerability of Alpelisib-resistant breast cancer cells is mediated by mTORC1

**Fig. 2:**
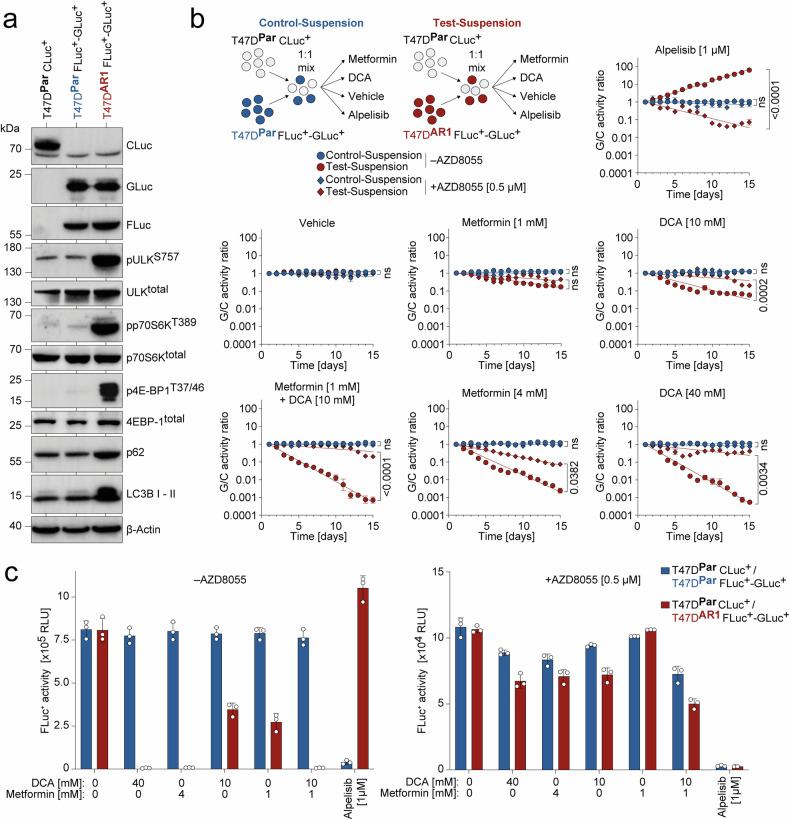
Metabolic vulnerability of Alpelisib-resistant breast cancer cells is mediated by mTOR. T47D^Par^ cells were labeled with either CLuc (T47D^Par^ CLuc^+^) or FLuc-GLuc (T47D^Par^ FLuc^+^-GLuc^+^) and T47D^AR1^ cells with FLuc-GLuc (T47D^Par^ FLuc^+^-GLuc^+^). **a** Western blot of luciferase-labeled T47D^Par^ and T47D^AR1^ cells. **b**, **c** Proliferation competition assay. For Control-Suspension T47D^Par^ CLuc^+^ and T47D^Par^ FLuc^+^-GLuc^+^ cells and for Test-Suspension T47D^Par^ CLuc^+^ and T47D^AR1^ FLuc^+^-GLuc^+^ cells were mixed in a 1:1 ratio, cultured 15 days in the presence or absence of AZD8055 (0.5 µM) in co-treatment with the indicated drugs and monitored daily for GLuc/CLuc activity (G/C activity) in the culture supernatant. **b** Shown is the G/C ratio normalized to day 1 ± SD, *n* = 3. FDR q values, one-way ANOVA with Tukey’s multiple comparisons test. **c** Endpoint measurement of intracellular FLuc activity within the Control- and Test-Suspension after 15-day treatment with indicated drugs in the presence or absence of AZD8055 (0.5 µM). Shown are mean ± SD, *n* = 3

The notable metabolic hypersensitivity to DCA/Metformin observed across distinct Alpelisib-resistant tumor cells, coupled with their concurrent resistance to the pan-PI3Ki Pictilisib, implies a mechanistic association between PI3Ki-resistance and metabolic susceptibility. As resistance is mechanistically driven by the selection of altered subclones, we further validated the association between PI3Ki-resistance and metabolic vulnerability in a competitive co-culture setting, using a dual-secreted luciferase assay for simultaneous monitoring of two differently labeled cell populations.^44^ To distinguish T47D^Par^ and T47D^AR1^ cells, they were labeled with the luciferase reporters Cyprindina luciferase (CLuc), Gaussia luciferase (GLuc), and Firefly luciferase (FLuc), which have distinct substrate specificities yielding T47D^Par^ CLuc^+^, T47D^Par^ FLuc^+^-GLuc^+^ and T47D^AR1^ FLuc^+^-GLuc^+^ labeled cells (Fig. 2a). Both CLuc and GLuc are actively secreted by the tumor cells, so that their concentration in the supernatant serves as a precise measure of viable tumor cells in a time-dependent manner. FLuc is an intracellular, non-secreted luciferase and was used for endpoint measurements.^44,45^ In competitive co-cultures of T47D^Par^ CLuc^+^ with T47D^AR1^ FLuc^+^-GLuc^+^ cells (Test-Suspension) or with T47D^Par^ FLuc^+^-GLuc^+^ cells (Control-Suspension) as reference, the ratio of GLuc to CLuc (G/C ratio) in the supernatant of the Test-Suspension progressively dropped in the presence of DCA/Metformin single treatment, indicating depletion of Alpelisib-resistant T47D^AR1^ FLuc^+^-GLuc^+^ cells from the co-culture in a dose-dependent manner whereas the G/C ratio remained stable in the Control-Suspension (Fig. 2b). Particularly, when combining DCA/Metformin treatment in lower concentrations (10 mM and 1 mM, respectively) a comparable efficacy could be achieved with a more than 100-fold depletion of T47D^AR1^ FLuc^+^-GLuc^+^ cells (Fig. 2b and Supplementary Fig. 1).

In line with the role of mTORC1 activation as the potential underlying cause of Alpelisib-resistance, T47D^AR1^ cells displayed strongly elevated mTORC1 signaling activity, indicated by phosphorylation of mTORC1 target sites (p70S6K^T389^, 4E-BP1^T37/46^ and ULK^S757^) (Fig. 2a). Importantly, the ATP-competitive mTOR kinase inhibitor AZD8055^46^ effectively resensitized T47D^AR1^ FLuc^+^-GLuc^+^ cells to Alpelisib, while simultaneously attenuating the response in the presence of Metformin/DCA (Fig. 2b). The observed changes in cellular abundance were also analyzed by measurement of intracellular firefly luciferase activity in cell lysates at the time course endpoint (Fig. 2c). Consistent with the time course analysis, the endpoint measurements confirmed that pharmacological mTOR inhibition with AZD8055 reversed resistance to Alpelisib but in parallel mitigated the response to metabolic drugs (Fig. 2c).

Notably, similar to the pan-mTOR kinase inhibitor AZD8055, the mTORC1-selective inhibitors Rapamycin and Everolimus also resensitized T47D^AR1^ and T47D^AR2^ cells to Alpelisib, yet rendered them resistant to metabolic drugs (Supplementary Fig. 2a, b). These findings confirm mTORC1 as the key mTOR kinase involved and underscore that, while both mTOR inhibitors and metabolic drugs effectively kill Alpelisib-resistant BC cells individually, they antagonize each other when combined.

We conclude that mTORC1 activation mechanistically links Alpelisib-resistance and metabolic susceptibility to DCA/Metformin.

### mTORC1-mediated autophagy defect and energy crisis in Alpelisib-resistant breast cancer cells

mTOR plays a central role in coordinating eukaryotic cell growth and metabolic responses to environmental stimuli. It regulates key cellular functions, including protein biosynthesis and autophagy, which are critical for cell survival during energy stress.^16,21^ This implies that the ability of mTOR to inhibit autophagy may contribute to the susceptibility of Alpelisib-resistant BC cells to DCA/Metformin treatment. Supporting this, T47D^AR1^ cells exhibited not only strongly elevated mTORC1 signaling activity but also impaired LC3B-I to II processing and increased p62/SQSTM1 levels, indicative of autophagy defects under basal conditions (Fig. 2a).

**Fig. 3:**
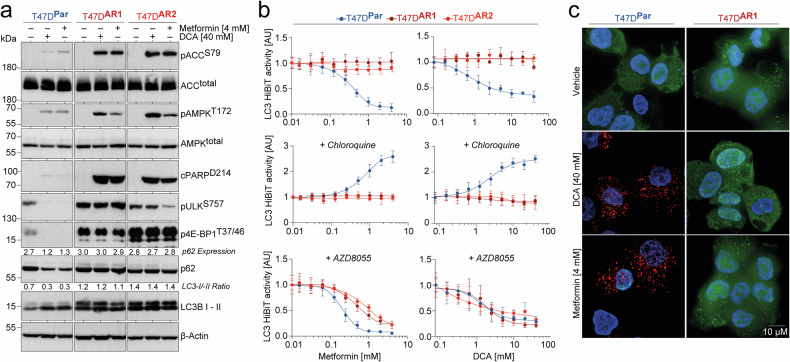
mTOR-mediated autophagy deficiency in Alpelisib-resistant breast cancer cells induces energy stress and apoptosis upon metabolic drug treatment. **a** Western blot of parental (T47D^Par^) and Alpelisib-resistant cells (T47D^AR1^, T47D^AR2^) treated with 40 mM DCA or 4 mM Metformin for 48 h as indicated. p62 and LC3B I - II levels were quantified by ImageJ and normalized to β-actin serving as loading control. The LC3B-I/-II ratio was calculated by dividing the values of LC3B-I and LC3B-II. **b** Autophagic flux analysis. LC3-HiBiT-expressing T47D^Par^, T47D^AR1^, and T47D^AR2^ cells were pre-treated as indicated with chloroquine (50 µM) or AZD8055 (0,5 µM) for 48 h and then treated with increasing doses of Metformin or DCA for 6 h. Shown is LC3 HiBiT reporter activity measured as luminescence normalized to untreated. Mean ± SD, *n* = 3. **c** Representative immunofluorescence images of DsRed-LC3-GFP-expressing T47D^Par^ and T47D^AR1^ cells following 48 h treatment with 40 mM DCA or 4 mM Metformin. Scale bars, 10 μM

In line with established metabolic effects, treatment with DCA/Metformin induced clear signs of energy stress in Alpelisib-resistant subclones T47D^AR1^ and T47D^AR2^. This was demonstrated by AMPK activation (pAMPK^T172^) and the inhibitory phosphorylation of its downstream target, acetyl-CoA carboxylase (pACC^S79^), the key enzyme of anabolic de novo fatty acid synthesis, ultimately leading to apoptosis as shown by PARP cleavage (Fig. 3a).^47^ Importantly, in Alpelisib-resistant subclones DCA/Metformin treatment had no detectable impact on p62 levels and LC3B processing (constant LC3B-I/LC3B-II ratio), demonstrating their failure to initiate autophagy in response to metabolic perturbation. In contrast, T47D^Par^ cells reacted to DCA/Metformin with induction of autophagy, evidenced by degradation of p62/SQSTM1 and increased LC3B processing (decreased LC3B-I/LC3B-II ratio), thereby bypassing severe energy stress and apoptosis (Fig. 3a).

Hypothesizing that the varied response of T47D^Par^ and Alpelisib-resistant subclones to DCA/Metformin reflects mTORC1-dependent differences in autophagic flux, we performed LC3 turnover assays.^48^ To precisely measure LC3 protein levels, we utilized a HiBiT-tagged LC3, which yields robust and quantitative luminescence via high-affinity interaction with NanoLuc.^49^ In support of an autophagy defect in Alpelisib-resistant BC cells, we observed a dose-dependent decrease in LC3 HiBiT levels under treatment with Metformin or DCA specifically in T47D^Par^ cells (Fig. 3b, upper panel). Recognizing that LC3 steady-state levels alone do not capture autophagic flux dynamics,^50^ we also assessed LC3 accumulation in the presence of chloroquine (Fig. 3b, middle panel). Chloroquine blocks LC3 degradation at the autolysosomal stage,^51^ so that LC3 accumulation in chloroquine-treated cells reflects upstream autophagic flux. Under these conditions, DCA/Metformin-treatment induced LC3 accumulation only in T47D^Par^ cells, highlighting the failure of T47D^AR1^ and T47D^AR2^ cells to properly upregulate autophagic flux upon metabolic stress. To clarify the role of mTOR signaling in this differential autophagic response, we repeated the experiments in the presence of the mTOR kinase inhibitor AZD8055. Notably, AZD8055 restored autophagic LC3 degradation in Alpelisib-resistant subclones, with no evident impact on T47D^Par^ cells (Fig. 3b, lower panel). This result confirms that mTOR activity attenuates the ability of T47D^AR1^ and T47D^AR2^ cells to increase autophagic flux in response to DCA/Metformin.

These findings were confirmed using cells expressing a DsRed-LC3-GFP tandem reporter. This reporter produces diffuse cytoplasmic green fluorescence that upon autophagy induction transitions to red-fluorescent puncta, representing autophagosomes.^52^ In accordance with the observed deficiency in T47D^AR1^ and T47D^AR2^ cells to elicit an autophagic response to DCA/Metformin treatment, only T47D^Par^ cells displayed a pronounced increase in red-fluorescent LC3 puncta upon treatment (Fig. 3c).

We conclude that autophagy mitigates lethal energy stress during metabolic perturbation, and that sustained mTORC1 signaling in Alpelisib-resistant BC cells increases their sensitivity to DCA/Metformin by disrupting this survival pathway.

### CRISPR/Cas9-engineered suppression of autophagy sensitizes to DCA/Metformin

**Fig. 4:**
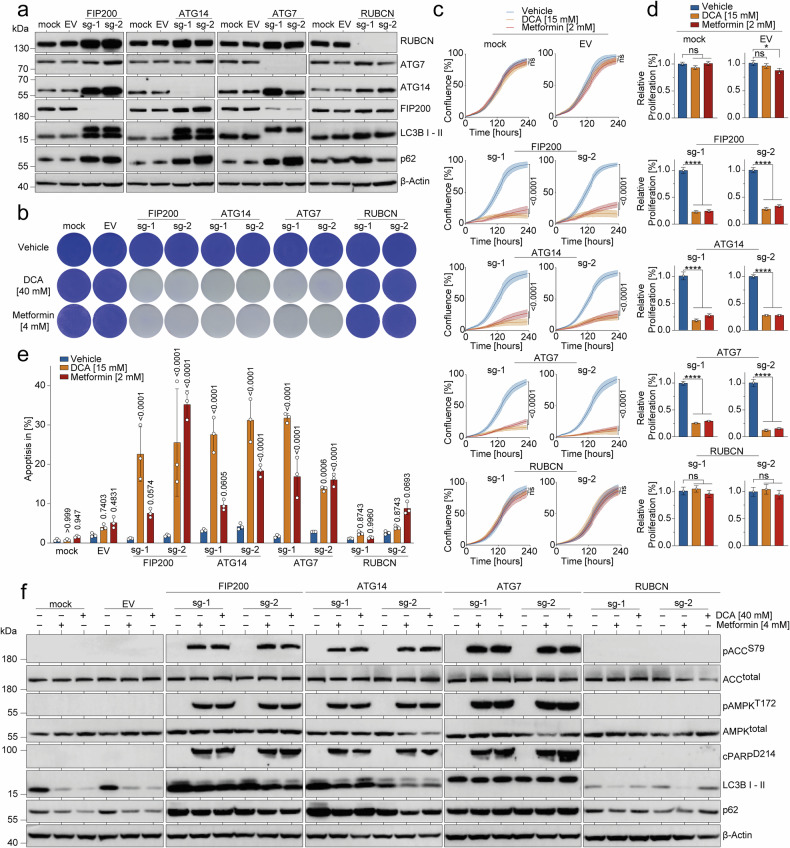
CRISPR/Cas9-engineered autophagy-deficiency in breast cancer cells induces metabolic vulnerability. **a**–**f** T47D^Par^ cells were infected with plentiCRISPRv2 vectors targeting *FIP200*, *ATG14*, *ATG7*, and *RUBCN* (sg-1 and sg-2, two independent sgRNAs per gene). After lentiviral transduction and puromycin selection, cells were single-cell cloned and examined for target gene knockout by Western blot (**a**). Mock: non-infected cells; EV: empty vector control. **b** Clonogenic growth of control (mock and EV) and knockout cells treated with DCA or Metformin. Shown are representative images. **c**, **d** Real-time live-cell imaging of control (mock and EV) and knockout cells treated with DCA or Metformin. **c** Mean confluence in % over time ± SD (*n* = 3). FDR *q* values. **d** AUC of proliferation curves relative to untreated. Shown is the mean ± SD (*n* = 3), one-way ANOVA with Dunnett’s multiple comparisons test, *p < 0.05; ****p < 0.0001. **e** Flow cytometry analysis for apoptosis (sub-G1). Indicated knockout cells were treated with DCA or Metformin for 5 days. Shown are mean ± SD, *n* = 3, two-way ANOVA with Tukey’s multiple comparisons test. **f** Western blot of control (mock and EV) and knockout cells treated with 40 mM DCA or 4 mM Metformin for 72 h

For a more thorough investigation into the protective function of autophagy amidst metabolic perturbation, we generated various T47D^Par^ cell clones with disruptive CRISPR/Cas9-induced insertion/deletion mutations in *FIP200*, *ATG14*, and *ATG7* – three genes essential for the canonical autophagy cascade. All knockout cell clones showed p62 accumulation and varying degrees of defects in LC3B-I and LC3B-II processing. Cells with a knockout of *RUBCN*, a gene involved in LC3-associated phagocytosis (LAP) but not in canonical autophagy, served as an internal specificity control and showed no alterations (Fig. 4a).^53–55^

Importantly, autophagy deficiency by knockout of *FIP200*, *ATG14*, or *ATG7*, but not *RUBCN*, rendered therapy-naïve T47D^Par^ cells sensitive to DCA/Metformin treatment, resulting in impaired clonogenic growth and proliferation (Fig. 4b–d). Similarly, as observed in PI3Ki-resistant cells, the knockout of essential autophagy genes caused T47D^Par^ cells to experience severe energy stress and initiate apoptosis upon DCA/Metformin treatment (Fig. 4e, f).

To validate these observations in a second BC model, we knocked out *FIP200*, *ATG14* and *ATG7* in MCF-7 cells, derived from a *PIK3CA*^E545K^ mutant HR^+^/Her2^−^ BC patient.^56^ In line with the results from T47D breast cancer cells, autophagy-deficient MCF-7 cells showed strongly diminished clonogenic growth and increased apoptosis when treated with DCA/Metformin. In contrast, parental and RUBCN-deficient MCF-7 cells did not respond to the treatment (Supplementary Fig. 3).

We concluded that autophagy suppression is sufficient for DCA/Metformin hypersensitivity in HR^+^/Her2^−^ breast cancer with *PIK3CA* mutations.

### Autophagy suppression exacerbates metabolic stress under metabolic drug treatment

**Fig. 5:**
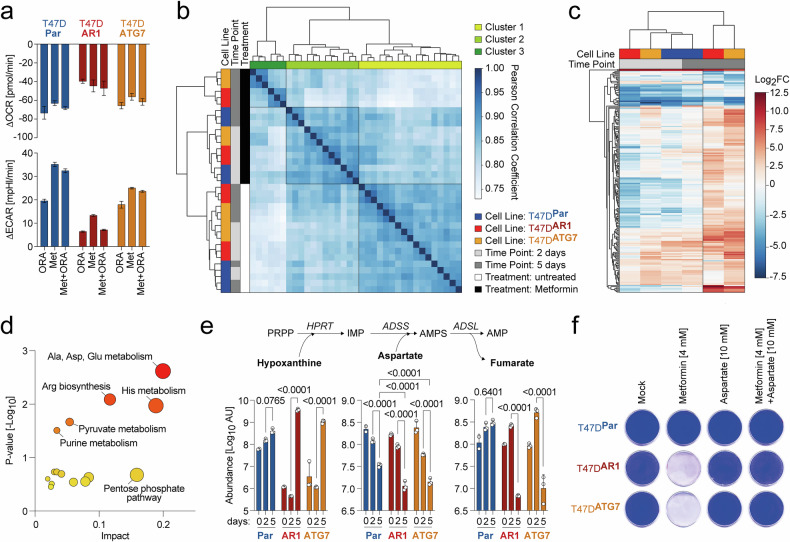
Autophagy-deficiency exacerbates metabolic stress induced by metabolic drugs. **a** Change in oxygen consumption rate (OCR, top) and extracellular acidification rate (ECAR, bottom) in response to treatment with Metformin and/or the combination of Oligomycin, Rotenone, Antimycin A (ORA). Data are presented as mean ± SD (*n* = 6). **b** Metabolomic profiles of untreated and Metformin-treated cells at 2 and 5 days post-treatment were compared in a pairwise correlation analysis. Shown is a heatmap of the Pearson correlation matrix, illustrating the similarity of metabolic profiles across conditions. Hierarchical clustering reveals distinct patterns of metabolic shifts over time and across different cell lines. **c** Hierarchically clustered heatmap of Metformin-induced metabolite changes (log2-fold) in the indicated cell lines 2 and 5 days post-treatment. **d** Pathway enrichment analysis (MetaboAnalyst 6.0) of metabolites altered by Metformin in T47D^AR1^ and T47D^ATG7^ cells after 5 days, compared to all other samples. **e** Cellular abundance of the purine salvage pathway components aspartate, hypoxanthine, and fumarate in the indicated untreated and Metformin-treated cells. Untargeted LC/MS metabolomics data are presented as mean ± SD (*n* = 3). Statistical significance was determined by two-way ANOVA with Tukey’s multiple comparisons test. PRPP phosphoribosyl pyrophosphate, HPRT hypoxanthine-guanine phosphoribosyltransferase, IMP inosine monophosphate, ADSS adenylosuccinate synthase, AMPS adenylosuccinate, ADSL adenylosuccinate lyase, AMP adenosine monophosphate. **f** Clonogenic growth assay demonstrating rescue of Metformin vulnerability by supplementation with 10 mM L-aspartate

To better understand the impact of autophagy suppression on cellular responses to metabolic drugs, we focused on Metformin due to its promising translational potential. Consistent with its role as an electron transport chain (ETC) inhibitor,^57,58^ a 6-h Metformin treatment of T47D^Par^, T47D^AR1^, and T47D ATG7-knockout (T47D^ATG7^) cells significantly reduced the oxygen consumption rate (OCR), comparable to the combined inhibition effects of Oligomycin, Rotenone, and Antimycin A (Fig. 5a). This decrease in OCR was accompanied by a compensatory increase in glycolytic flux, measured as extracellular acidification rate (ECAR), which also matched the shift observed with the combined ETC inhibitors.

To further investigate the metabolic consequences, we performed untargeted LC-MS metabolomics on untreated and Metformin-treated T47D^Par^, T47D^AR1^, and T47D^ATG7^ cells at 2 and 5 days post-treatment, with three biological replicates per condition. Hierarchical clustering and principal component analysis revealed tight clustering of untreated samples, indicating minimal baseline metabolic variation among cell types (Fig. 5b, Supplementary Fig. 4a). After 5 days of treatment, autophagy-competent T47D^Par^ samples clustered closely with the 2-day-treated samples, whereas autophagy-deficient T47D^AR1^ and T47D^ATG7^ cells clustered distinctly from both untreated and 2-day-treated samples, highlighting substantial and progressive metabolic shifts. These observations were further confirmed by a heatmap of Metformin-induced metabolic changes (Fig. 5c). While all three cell types initially exhibited a modest and similar response to Metformin, the response intensified in the T47D^AR1^ and T47D^ATG7^- cell types over time. These findings support a critical role for autophagy in buffering the metabolic stress induced by Metformin, suggesting that autophagy deficiency heightens sensitivity to Metformin’s metabolic effects over prolonged treatment durations.

To identify specific metabolic pathways that may underlie the reduced proliferation and viability of autophagy-deficient cells under Metformin treatment, we analyzed metabolites that were differentially expressed in 5-day-treated T47D^AR1^ and T47D^ATG7^ samples relative to all others using MetaboAnalyst 6.0. This analysis highlighted the ‘Alanine, aspartate, and glutamate metabolism’ pathway as the most significantly altered (Fig. 5d). Metformin-induced ETC inhibition limits the availability of electron acceptors and reduces the NAD^+^/NADH ratio, essential for aspartate biosynthesis – a pathway frequently constrained in cancer cells due to limited aspartate availability or uptake.^58–62^ While aspartate levels declined across all Metformin-treated samples, the reduction was significantly more pronounced and progressive in autophagy-deficient cells (Fig. 5e, Supplementary Fig. 4c), potentially reaching critically low levels.

Aspartate is essential for purine biosynthesis through both de novo and salvage pathways.^63^ Given the high energy requirements of de novo synthesis, glycolytic cells, such as red blood cells, neurons, and many cancer cells, heavily depend on the salvage pathway, especially under low-energy conditions.^63,64^ In this pathway, hypoxanthine is converted to AMP in an aspartate-consuming, fumarate-producing reaction cascade (Fig. 5e). Consistent with a critical aspartate depletion in autophagy-deficient cells, we observed a >1000-fold increase in hypoxanthine accompanied by a marked decrease in fumarate (Fig. 5e). Confirming a causal role of aspartate depletion, supplementation with aspartate effectively rescued the initially vulnerable Alpelisib-resistant and genetically engineered autophagy-deficient cells from Metformin-induced cell death, confirming that the observed aspartate depletion was the root cause of Metformin sensitivity (Fig. 5f, Supplementary Fig. 4b, d, e). Collectively, these findings underscore the essential role of autophagy in counteracting Metformin-induced aspartate depletion, highlighting that Alpelisib-resistant, autophagy-deficient breast cancer cells are particularly vulnerable to Metformin due to their inability to maintain adequate aspartate levels in vitro.

### Metabolic inhibitors selectively target Alpelisib-resistant breast cancer in vivo

**Fig. 6:**
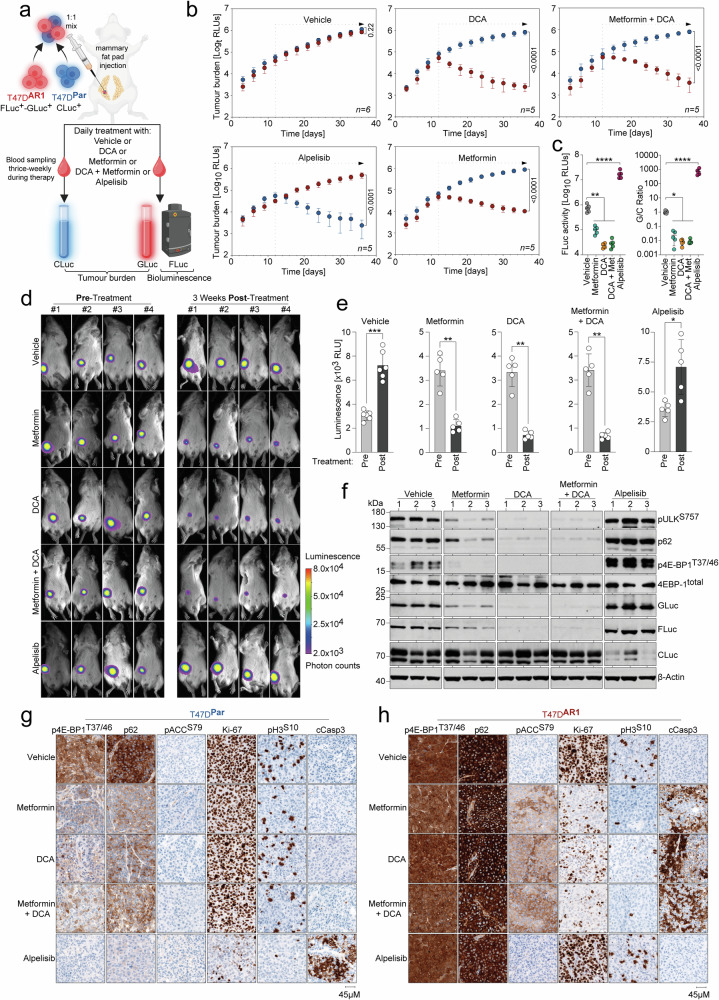
Metabolic drugs selectively target Alpelisib-resistant breast cancer cells in vivo. **a**–**h** Mice were orthotopically injected in the mammary fat pad with a 1:1 ratio of T47D^Par^ CLuc^+^ and T47D^AR1^ GLuc^+^-FLuc^+^ cells labeled with the secreted luciferases CLuc and GLuc, respectively, and treated as indicated. FLuc remains intracellular and was used for BLI. Tumor burden was quantified separately for each cell type by longitudinal CLuc and GLuc activity measurement in blood samples. **b** Tumor growth curves show the tumor burden (RLU, relative light units) over time as mean ± SD of *n* = 6 mice in the vehicle cohort and *n* = 5 mice in each treatment cohort, FDR *q* values. **c** FLuc activity and G/C Ratio of tumor lysates in (**a**). FLuc activity and G/C Ratio in tumor lysates were tested for statistical significance by two-way ANOVA with Dunnett’s multiple comparisons test. *p*-values denote pairwise comparisons of treatment cohorts with vehicle *p < 0.05; **p < 0.01; ****p < 0.0001. **d** Exemplary bioluminescence imaging (BLI) pictures of representative mice from each cohort in (**a**) before and after three weeks of treatment. **e** Luminescence quantification using Bruker Multiplex Software in a defined region of interest (ROI) of all mice in (**a**). Shown is the mean luminescence intensity ± SD. Statistical significance was tested using unpaired Mann–Whitney U test: *p < 0.05; **p < 0.01; ***p < 0.001. **f** Western blot of 3 tumors from each mouse cohort in (**a**). **g**, **h** T47D^Par^ and T47D^AR1^ cells were injected into the mammary fat pad of immunodeficient mice. Developing tumors were treated with the indicated drugs for 3 days. Tissue sections were immunostained for the indicated proteins. Shown are representative images of tumors from each treatment cohort. Scale bars, 45 μM

We proceeded to evaluate the efficacy of metabolic compounds for targeting breast cancer with mTORC1-dependent PI3Ki-resistance in vivo. To directly compare the effects of metabolic inhibitors on therapy-naïve and Alpelisib-resistant tumor cells, we orthotopically injected a 1:1 mixture of T47D^Par^ CLuc^+^ and T47D^AR1^ FLuc^+^-GLuc^+^ cells into the mammary fat pad of immunodeficient mice.^65^ This approach, previously used for competitive co-culture luciferase assays (Fig. 2a), allowed us to monitor tumor cell dynamics in vivo. GLuc and CLuc are actively secreted by tumor cells, and their activity levels in blood samples accurately reflect the viable tumor mass within the organism.^44^ Thus, luciferase activity measurements of blood samples collected at regular time intervals during therapy provided cell-type-specific longitudinal monitoring of tumor burden in a competitive setting (Fig. 6a).^44^ FLuc, used to label T47D^AR1^ tumors, is retained within the tumor cells, allowing for the evaluation of therapeutic efficacy on Alpelisib-resistant cells through bioluminescence imaging (BLI) (Fig. 6a).

All animals were treated daily during the week with either DCA, Metformin, or a combination of DCA and Metformin for three weeks starting two weeks after tumor cell injection. Two control cohorts received either Alpelisib or a vehicle. While the growth of *PIK3CA*^mut^ T47D^Par^ tumors was effectively inhibited by Alpelisib treatment, T47D^AR1^ tumor growth expectedly remained unaffected. Vice versa, Metformin and DCA selectively inhibited T47D^AR1^ tumor growth, resulting in a 100-fold reduction of Alpelisib-resistant cells in the end-stage tumors within the Metformin cohort and a 1000-fold reduction in the DCA cohort with no additional effect when combining both drugs (Fig. 6b, c). Likewise, FLuc-based BLI of T47D^AR1^ tumors demonstrated continuous tumor growth in vehicle and Alpelisib-treated mouse cohorts, but significantly decreased tumor burden after three weeks of metabolic drug treatment, confirming the strong metabolic vulnerability of T47D^AR1^ tumors (Fig. 6d, e).

To validate these observed alterations, tumor lysates from end-stage mice were analyzed for luciferases, mTORC1 and autophagy markers (Fig. 6f). Compared to breast tumors from untreated mice, which contained both tumor cell types, tumors from the Alpelisib-treated cohort displayed upregulation of mTORC1 signaling, defects in p62 degradation, increased FLuc and GLuc and reduced CLuc level, consistent with the Alpelisib-induced enrichment of T47D^AR1^ FLuc^+^-GLuc^+^ and negative selection of T47D^Par^ CLuc^+^ cells (Fig. 6b–e). In contrast, breast tumors from DCA/Metformin-treated mice showed decreased mTORC1 signaling, reduced levels of p62, and decreased FLuc and GLuc expression (Fig. 6f), consistent with the depletion of T47D^AR^ cells by metabolic drugs. Thus, the proliferation of Alpelisib-resistant T47D^AR1^ tumor cells was selectively inhibited by DCA/Metformin therapy in vivo, leading to the effective elimination of Alpelisib-resistant cells from heterogeneous breast tumors.

For a detailed examination of the acute in vivo response to metabolic inhibitors, cohorts of mice harboring either T47D^Par^ or T47D^AR1^ breast tumors were subjected to three-day treatments with DCA and Metformin, administered either separately or in combination, with the vehicle and Alpelisib treatment serving as a control. Immunohistochemical analysis of tumor sections revealed elevated phosphorylation of 4E-BP1^T37/46^ in untreated T47D^AR1^ tumors compared to T47D^Par^ tumors, in line with upregulated basal mTORC1 signaling in Alpelisib-resistant tumors in vivo. Notably, mTORC1 signaling in T47D^Par^ tumors decreased following treatment, while it persisted in T47D^AR1^ tumors not only under metabolic drug but even under Alpelisib treatment (Fig. 6g, h).

Metabolic drug treatment of T47D^Par^ tumors induced p62 degradation, indicating autophagy induction.^50^ Contrarily, T47D^AR1^ tumors demonstrated sustained elevation in p62 levels following metabolic drug treatment. This was accompanied by increased ACC^S79^ phosphorylation, indicative of pronounced energy stress. As a result, metabolically treated T47D^AR^ tumors exhibited decreased proliferation and enhanced apoptosis, as indicated by reduced Ki67/H3^S10^ and increased cleaved Caspase-3 staining, respectively (Fig. 6g, h).

This underscores the incapacity of PI3Ki-resistant breast tumors with elevated mTORC1 signaling to initiate autophagy in response to pharmacologically induced energy stress, rendering them particularly susceptible to targeting by metabolic drugs in vivo.

### Metabolic inhibitors selectively target *ATG7*-knockout breast cancer in vivo

To determine if autophagy deficiency alone is sufficient to induce metabolic vulnerability for targeted in vivo treatment, we utilized the CRISPR/Cas9-engineered *ATG7*-knockout cells (T47D^ATG7^), as ATG7 is essential for the key degradative stages of the canonical autophagy cascade.^66^ As described in the previous sections (Figs. 2 and 6), we measured the drug responses in a competitive setting using the dual-secreted luciferase assay with T47D^Par^ CLuc^+^, T47D^Par^ FLuc^+^-GLuc^+^, and T47D^ATG7^ FLuc^+^-GLuc^+^ cells. Confirming their genetically engineered autophagy defect, T47D^ATG7^ FLuc^+^-GLuc^+^ cells showed p62 accumulation and failure of LC3-I to LC3-II processing (Supplementary Fig. 5a). In co-cultures of T47D^Par^ CLuc^+^ with either T47D^ATG7^ FLuc^+^-GLuc^+^ (Test-Suspension) or T47D^Par^ FLuc^+^-GLuc^+^ cells (Control-Suspension), the *ATG7*-knockout tumor cells were progressively depleted by DCA and Metformin, confirming their metabolic vulnerability in a competitive setting in vitro (Supplementary Fig. 5b–d).

**Fig. 7:**
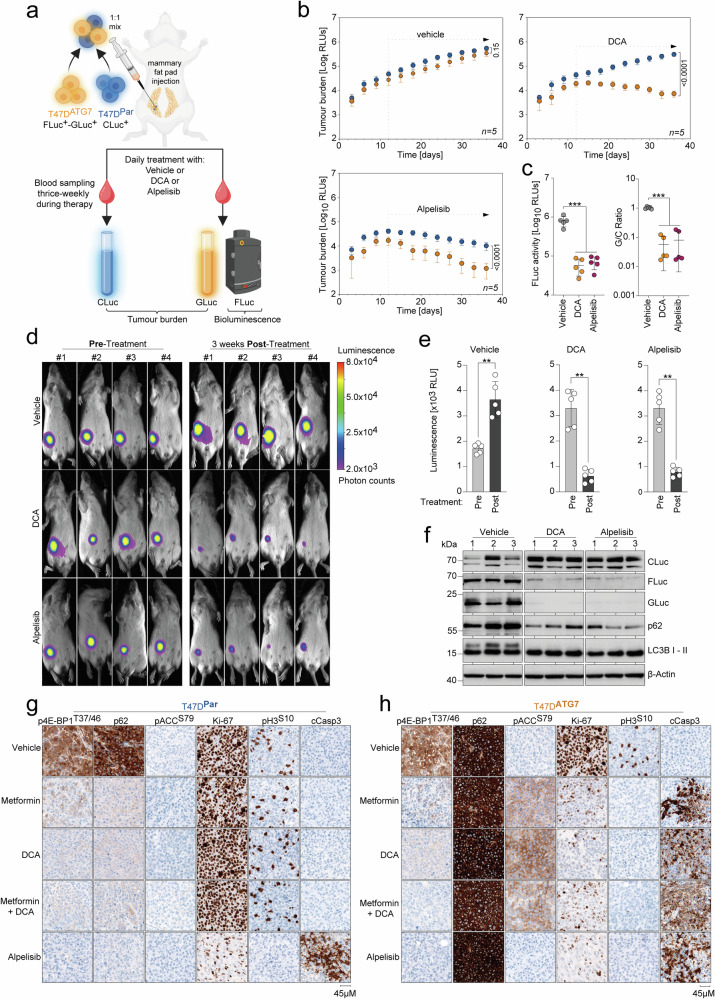
Metabolic drugs selectively target autophagy-deficient breast cancer cells in vivo. **a**–**h** Mice were orthotopically injected in the mammary fat pad with a 1:1 ratio of T47D^Par^ CLuc^+^ and T47D^ATG7^ GLuc^+^-FLuc^+^ cells labeled with the secreted luciferases CLuc and GLuc, respectively, and treated as indicated. FLuc remains intracellular and was used for BLI. Tumor burden was quantified separately for each cell type by longitudinal CLuc and GLuc activity measurement in blood samples. **b** Tumor growth curves show the tumor burden over time as mean ± SD of *n* = 5 mice in each cohort, FDR *q* values. **c** FLuc activity and G/C Ratio of tumor lysates in (**a**). FLuc activity and G/C Ratio in tumor lysates were tested for statistical significance by two-way ANOVA with Dunnett’s multiple comparisons test. *p*-values denote pairwise comparisons of treatment cohorts with vehicle ***p < 0.001. **d** Exemplary bioluminescence imaging (BLI) pictures of representative mice from each cohort in (**a**) before and after three weeks of treatment. **e** Luminescence quantification using Bruker Multiplex Software in a defined region of interest (ROI) of all mice in (**a**). Shown is the mean luminescence intensity ± SD. Statistical significance was tested using unpaired Mann–Whitney U test: **p < 0.01. **f** Western blot of 3 tumors from each mouse cohort in (**a**). **g**, **h** T47D^Par^ and T47D^ATG7^ cells were injected into the mammary fat pad of immunodeficient mice. Developing tumors were treated with the indicated drugs for 3 days. Tissue sections were immunostained for the indicated proteins. Shown are representative images of tumors from each treatment cohort. Scale bars, 45 μM

Given that in the previous in vivo trial, DCA demonstrated the highest therapeutic efficacy against PI3Ki-resistant cells when used as a single agent (Fig. 6b), we proceeded to test the response of *ATG7*-knockout cells to DCA in the orthotopic breast cancer xenograft model, following the same experimental design (Fig. 7a). Importantly, *ATG7*-deficient tumors were highly susceptible to DCA, whereas parental autophagy-competent tumors did not respond (Fig. 7b). Notably, *ATG7*-knockout also increased the sensitivity of tumors to Alpelisib, suggesting autophagy deficiency as a possible sensitizing mechanism for PI3Ki treatment (Fig. 7b–e). Analysis of end-stage tumors further validated that both DCA and Alpelisib treatment depleted T47D^ATG7^ FLuc^+^-GLuc^+^ autophagy-deficient cells and selected for autophagy-competent CLuc^+^ parental cells, confirming autophagy deficiency as a sensitizing mechanism for both treatments (Fig. 7f).

To investigate the acute in vivo response of *ATG7*-knockout tumors to metabolic drugs and Alpelisib, cohorts of mice bearing either T47D^Par^ or T47D^ATG7^ cells underwent three-day drug treatments. Immunohistochemical analysis of tumor sections revealed higher p62 levels in untreated T47D^ATG7^ tumors compared to T47D^Par^ tumors, along with comparable phosphorylation levels of 4E-BP1^T37/46^, confirming the genetically engineered autophagy defect independent of mTORC1 signaling in vivo. Importantly, metabolic drug and Alpelisib treatment both reduced mTORC1 signaling in T47D^Par^ and T47D^ATG7^ tumors, while p62 levels remained elevated exclusively in T47D^ATG7^ tumors, confirming the persistent mTORC1-independent autophagy defect under metabolic stress and PI3Ki treatment (Fig. 7g, h). Importantly, metabolic drug treatment induced severe energy stress, leading to reduced proliferation and increased apoptosis specifically in T47D^ATG7^ tumors. In contrast, Alpelisib treatment triggered a potent apoptotic response in both parental and *ATG7*-knockout tumors without evident signs of energy stress (Fig. 7g, h).

Notably, L-aspartate quantification using FFPE-fixed tumor tissue from the experiments shown in Fig. 6g, h and Fig. 7g, h after three days of Metformin treatment demonstrated a significant reduction in L-aspartate levels in Metformin-treated Alpelisib-resistant and *ATG7*-knockout tumor-bearing mice compared to parental tumors (Supplementary Fig. 4f), strongly suggesting that autophagy-deficient BC cells undergo apoptosis also in vivo due to their inability to maintain adequate L-aspartate levels.

### 4E-BP1^T37/46^ phosphorylation and p62 accumulation correlate and predict overall survival in breast cancer patients

**Fig. 8:**
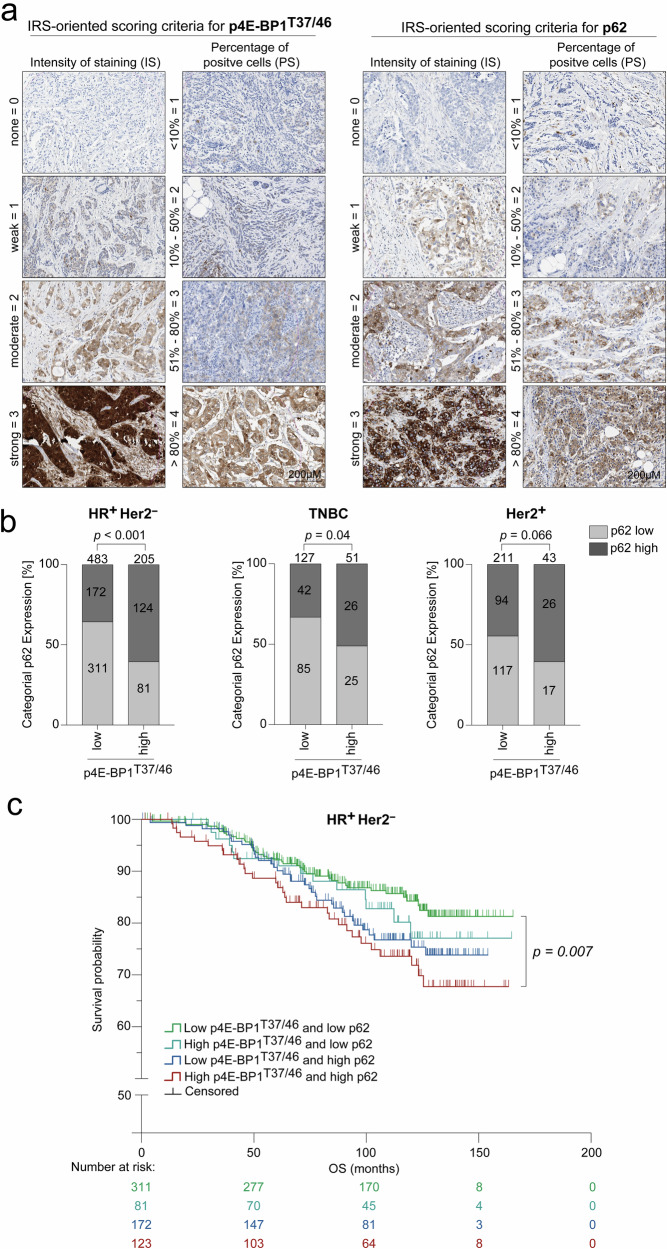
4E-BP1^T37/46^ phosphorylation and p62 accumulation correlate and together predict the overall survival of breast cancer patients. **a** p4E-BP1^T37/46^ and p62 tissue microarray (TMA) evaluation using IRS scoring. TMAs of paraffin-embedded breast cancer patient samples (*n* = 1120) were immunostained for p4E-BP1^T37/46^ and p62. Shown are representative immunostaining patterns indicating the intensity of staining (IS) and the percentage of positive cells (PS), graded from 0 (none) to 3 (strong) for IS and from <10% (1) to >80% (4) for PS. The IRS-oriented score for p4E-BP1^T37/46^ and p62 was determined by multiplying IS and PS. Scale bars: 200 μM. **b** p4E-BP1^T37/46^ and cytoplasmic p62 levels were evaluated using IRS scoring, calculated with VM Slide Explorer 2.2. Cutoff values (IRS Score 5) for classifying low (IRS < 5) and high (IRS > 5) 4E-BP1^T37/46^ and p62 expression were determined via Cutoff Finder. The correlation between p4E-BP1^T37/46^ and p62 is illustrated across indicated breast cancer subtypes (HR^+^/Her2^−^ (*n* = 688), TNBC (*n* = 178) and Her2^+^ (*n* = 254)). *P*-values were determined using a two-sided Fisher’s exact test, with significance defined as <0.05. **c** Kaplan–Meier survival analysis for indicated levels of p4E-BP1^T37/46^ and p62 (low: IRS < 5 or high: IRS > 5). Shown is the OS of HR^+^/Her2^−^ breast cancer patients (*n* = 687) according to p4E-BP1^T37/46^ and p62 levels. Significance was determined using a log-rank test: *p* = 0.050 (across all 4 arms), *p* = 0.007 (pairwise comparison of double-low (green) versus double-high (red) arms)

To investigate the presence of mTORC1-created autophagy defects in breast cancer patients as a potential exploitable metabolic vulnerability, we conducted tissue microarray (TMA) analyses on a cohort of 1120 early-stage node-positive primary breast cancer patients (Supplementary Fig. 6).^67^ The samples were stained for p4E-BP1^T37/46^ as a marker of mTORC1 signaling activity and for p62 as an indicator of autophagic degradation (Fig. 8a).^68^ For standardized evaluation of immunohistochemical data, we utilized the immunoreactive score (IRS) ranging from 0 to 12 calculated by multiplying staining intensity (0–3) by the percentage of positive cells (0–4), with an IRS of 5 identified as the optimal cutoff for binary classification of p4E-BP1^T37/46^ and p62 in low (**<**IRS 5) and high (**>**IRS 5) using the Cutoff Finder tool.^69,70^ Importantly, p4E-BP1^T37/46^ staining significantly correlated with p62 in HR^+^/Her2^−^ and triple-negative breast cancer (TNBC) patients suggesting an mTORC1-dependent autophagy deficiency in (124/688, 18%) of HR^+^/Her2^−^ and (26/178, 14.6%) of TNBC patients, while the association reached no significance in the Her2^+^ subtype (Fig. 8b). To determine whether p4E-BP1^T37/46^ and p62 accumulation function as prognostic biomarkers, we focused on the HR^+^/Her2^−^ subtype and found significant differences in survival among subgroups defined by the combination of p4E-BP1^T37/46^ and p62, with the worst OS observed in tumors exhibiting high levels of both markers (Fig. 8c).

We concluded that 4E-BP1^T37/46^ phosphorylation correlates with p62 accumulation in primary HR^+^/Her2^−^ and triple-negative breast cancer patients and that combined high levels of p4E-BP1^T37/46^ and p62 are associated with reduced OS in the HR^+^/Her2^−^ subgroup, thereby serving as prognostic biomarkers.

## Discussion

Characteristics of cancer metabolism, such as increased metabolic flux through intermediary metabolism to sustain proliferation, are primarily a consequence of alterations in signal transduction networks, such as PI3K-AKT-mTORC1, rather than direct mutations in metabolic enzymes.^33,71^ Given the broad range of targets in metabolic cancer therapy, there is a critical need to identify biomarkers that predict metabolically sensitizing alterations, facilitating patient stratification for targeting metabolic pathways in parallel with SoC treatments within a personalized medicine framework.^39^

In our study, we have revealed that constitutive upregulation of mTORC1 in Alpelisib-resistant breast cancer cells (Fig. 2a) leads to significant metabolic vulnerabilities, making them highly susceptible to glycolysis and mitochondrial ETC inhibitors both in vitro and in vivo (Fig. 1a–e and Fig. 6b–e). Metabolomic profiling of the Metformin response revealed depletion of aspartate (Fig. 5e), as reported previously.^58^ However, while Metformin-treated parental BC cells were able to maintain aspartate at a level compatible with cell survival and proliferation, aspartate dropped to critically low levels in Alpelisib-resistant cells as evidenced by massive alterations in metabolites of the purine salvage pathway. Consistent with the role of autophagy in macromolecular degradation, *ATG7*-knockout cells showed similar metabolic alterations (Fig. 5). Importantly, aspartate supplementation rescued cells from the cytotoxic effects of Metformin treatment, indicating that the aspartate depletion is responsible for the metabolic vulnerability of Alpelisib-resistant and autophagy-deficient BC cells. Moreover, orthotopic BC xenograft models confirmed that Alpelisib-resistant and *ATG7*-knockout cells are similarly susceptible to DCA and Metformin treatment in vivo whereas parental cells do not respond (Fig. 7b–e). This underlines the important role of autophagy in promoting the survival of *PIK3CA*^mut^ BC cells under metabolic perturbation while simultaneously presenting a druggable metabolic vulnerability.^72^ However, the clinical applicability of metabolically active drugs remains constrained by the inability to stratify eligible cancer patients due to the absence of reliable biomarkers and by the toxic side effects of many of these compounds.^34^

Based on our mechanistic findings, we evaluated the significance of p4E-BP1^T37/46^ and p62 as biomarkers, which reliably indicated mTORC1-mediated autophagy deficiency in Alpelisib-resistant (Fig. 6g, h) and mTORC1-independent autophagy defects in CRISPR-Cas9-engineered *ATG7*-knockout BC xenografts (Fig. 7g, h) via immunohistochemical analysis. In a cohort of over 1000 patients with lymphogenously metastasized BC, biomarker validation revealed a significant correlation between p4E-BP1^T37/46^ and p62 in HR^+^/Her2^−^ BC (Fig. 8b). This highlights a substantial subgroup of patients (high p4E-BP1^T37/46^ and high p62) with markedly worse OS (Fig. 8c). These findings validate p4E-BP1^T37/46^ in combination with p62 as a prognostic biomarker and suggest a potential therapeutic benefit of metabolic drugs in these patients due to mTORC1-mediated autophagy deficiency.

However, in clinical practice, core needle biopsies of the breast or metastatic lesions upon disease progression under Alpelisib treatment are uncommon, thereby limiting the utility of IHC-based stratification for metabolic drug treatment. Therefore, it is advisable to measure the expression of biomarkers such as p4E-BP1^T37/46^ and p62 across the entire resected breast tumor specimen post-operation and to consider combining metabolic therapy with adjuvant SoC treatments.^39^ In this light, the presence of organic cation transporters (OCTs) on breast tumor cells is crucial for Metformin’s cellular uptake due to its hydrophilic nature.^73^ In a xenograft mouse model of BC, overexpressing OCT3 in MCF-7 cells resulted in a three-fold reduction in tumor size compared to parental MCF-7 cells, indicating increased sensitivity to Metformin.^74^ This finding may support a rationale for combined screening of OCT3 and p4E-BP1^T37/46^/p62 expression in tumors to identify Metformin-responsive BC patients.^75^ However, beyond molecular aspects, various factors such as menopausal status, metabolic health, and tumor subtype could influence clinical outcomes in BC patients treated with Metformin, with growing evidence suggesting that HR^+^/Her2^−^ BC patients are the most suitable candidates for this option of metabolic therapy.^75,76^

Furthermore, it has been demonstrated that suppressing insulin feedback with a ketogenic diet, Metformin, or inhibitors of sodium-glucose co-transporter 2 (SGLT2) enhances the efficacy of PI3Ki like Alpelisib, Taselisib and Buparlisib in animal trials.^77^ In particular, early hyperglycemia during PI3Ki treatment resulting from PI3K-α inhibition impairs insulin’s metabolic actions, leading to increased blood glucose levels and treatment-induced hyperinsulinemia that may promote BC cell survival and reduce PI3Ki efficacy in several clinical studies.^78,79^ Therefore, it was a groundbreaking discovery that in syngeneic allograft models of *Pik3ca*-mutant (p.H1047R) murine BC, a ketogenic diet enhances the responses to Alpelisib by lowering blood insulin levels and consequently reducing insulin’s ability to activate the insulin receptor in tumors.^77^ However, it remains unclear whether combined Metformin and Alpelisib treatment enhances the tumor response in BC patients, as studies have primarily focused on the effectiveness of Metformin in managing Alpelisib-induced hyperglycemia rather than on the combined effect on tumor response.^80–82^

Despite correlating significantly in HR^+^/Her2^−^ and TNBC, no association of p4E-BP1^T37/46^ and p62 was observed in Her2^+^ BC patients (Fig. 8b). Based on evidence showing that endogenous Her2 promotes BC tumorigenesis by interacting with Beclin 1,^83^ it is conceivable that Her2 in those cells inhibits autophagy independently of PI3K-mTOR signaling.^83,84^ However, considering mTOR’s significant role in autophagy inhibition regardless of Her2 signaling, we demonstrated in non-small cell lung cancer (NSCLC) that mTOR-dependent upregulation of FancD2 protects from the cytotoxic DNA crosslinks induced by cisplatin, while in parallel inhibiting autophagy, thereby creating a druggable metabolic vulnerability in otherwise chemo-refractory NSCLC patients and highlighting mTOR as a double-edged sword in cancer therapy.^41,85,86^

Autophagy defects in cancer cells may not only be exploited by treatment with metabolic drugs like DCA or Metformin. In fact, many preclinical and clinical studies of pharmacological autophagy inhibitors were shown to enhance tumor cell death in combination with radiotherapy, chemotherapy, and other frequently employed molecular targeted agents in breast cancer, including CDK4/6i, PI3Ki, and AKTi.^87,88^ Importantly, we observed strong autophagy induction in therapy-naïve BC cells following metabolic drug treatment with Metformin or DCA, suggesting that the efficacy of Alpelisib could be compromised when directly combined with metabolic drug treatment. Furthermore, due to its ability to provide a plethora of metabolites to sustain metabolism, autophagy may mitigate the antitumor effects of diverse metabolic inhibitors, suggesting that the susceptibility of BC cells with genetically engineered or mTOR-induced autophagy defects might extend beyond metabolic drugs targeting glycolysis or mitochondrial ETC.^89^

Although essential canonical autophagy genes are not frequently affected by somatic point mutations, haploinsufficiency network analyses have revealed monoallelic deletions in *BECN1* in up to ∼30% of BC patients.^90,91^ This indicates that the autophagy status could potentially be inferred from genome sequencing data, however, the suitability of monitoring of ATG gene expression as a general readout for autophagy is not recommended, since most of the ATG genes do not show significant changes in mRNA levels when autophagy is induced.^50,92^ Given the lack of methods available to monitor autophagy in humans, IHC currently appears to be the best solution for identifying breast cancer patients with a functional autophagy defect suitable for metabolic therapy.

In summary, our study reveals that mTORC1 upregulation, a common cause of Alpelisib-resistance in BC, creates vulnerabilities to metabolically active drugs by suppressing autophagy and, thereby, depriving tumors of a central protective mechanism to endure metabolic stress. Importantly, biomarker analysis in BC patients identified the combination of p4E-BP1^T37/46^ and p62 as a biomarker for poor survival, suggesting a potential utility in identifying a prognostically challenging subset of BC patients with mTORC1-mediated autophagy defects that may profit from metabolic therapies.

## Methods

### Cell culture

Cell lines (T47D, MCF-7, HEK293T and Platinum-E cells) were obtained from the American Tissue Collection Center (ATCC) and grown in high-glucose DMEM supplemented with 10% fetal bovine serum, 100 U ml^−1^ penicillin, 100 μg ml^−1^ streptomycin, and 0.4% Amphotericin B (250 μg mL^−1^) at 37 °C with 5% CO_2_. All cell lines were authenticated by STR profiling and tested negative for mycoplasma contamination. Metabolic drugs and inhibitors were obtained from Sigma-Aldrich unless indicated otherwise and used at the following concentrations: Alpelisib (Selleckchem) 5 nM–2.5 µM, Pictilisib (Selleckchem) 1 µM, Dichloroacetate 10–40 mM, 2-DG 20 mM, Metformin 1–4 mM, Phenformin 125 µM, AZD8055 (Selleckchem) 0.5 µM, Chloroquine 25–100 µM. Standard concentrations for clonogenic growth assays were 40 mM DCA and 4 mM Metformin. Rescue experiments were performed at a final concentration of 10 mM L-Aspartate (A7219, Sigma-Aldrich).

Alpelisib-resistant cells were generated by dose escalation from 5 nM to 2.5 µM of Alpelisib and maintained under continuous treatment with 1 µM Alpelisib. Prior to the experiment, the resistant cells were cultured without Alpelisib for a minimum of one week.

### Cell viability assays

Cell viability in response to the treatment was evaluated using the CellTiter-Glo assay (Promega) according to the manufacturer’s protocol. Briefly, cells were seeded in white-walled 96-well plates overnight and 3 replicate wells were treated with inhibitors diluted in 80 µl medium the next day. After 5 days of treatment, 80 µl CellTiter-Glo reagent was added and luminescence was measured using an Orion II luminometer (Berthold). Background signal from empty wells was subtracted and luminescence was normalized to untreated control wells. Dose-response curves were fitted to the data points with GraphPad Prism software (inhibitor vs. response - variable slope four-parameter model) and used to determine the 50% inhibitory concentration (IC50) with 95% confidence intervals.

### Clonogenic growth assays

Cells were seeded overnight, treated, and cultured for 10 days with medium changes. Afterward, plates were fixed in 70% ethanol overnight, stained with a 1:20 diluted crystal violet solution (Sigma-Aldrich HT90132) in 20% ethanol for 30 min, washed with tap water, and air-dried.

### Live-cell imaging

Tumor cell proliferation was continuously monitored using the IncuCyte S3 Live-Cell Analysis System (Sartorius). Cells were seeded on 96-well plates overnight and subjected to metabolic drug treatment the following day. At intervals of 2 h, four phase-contrast images were captured per well at 10× magnification, with each treatment condition replicated across three wells. Confluence analysis was performed with IncuCyte S3 2018A software in Phase Object Confluence mode, using a segmentation score of 0.7 and excluding objects smaller than 500 μm^2^. Proliferation was measured as the area under the confluence curve (AUC) using GraphPad Prism (version 10.2.3).

### Apoptosis analysis

Cell culture supernatant was collected, and adherent cells were detached using trypsin. They were then combined with the supernatant, washed with PBS, and suspended in 1 ml PBS. After fixing with 10 ml ice-cold 90% ethanol, cells were stained with propidium iodide (10 µg ml^−^^1^) and RNase A (100 µg ml^−^^1^). Subsequently, sub-G1 content was analyzed using Accuri C6 Plus (BD Bioscience) or Cytoflex (Beckman) flow cytometer.

### LC3 HiBiT autophagy assays

For the analysis of autophagic flux, the autophagy LC3 HiBiT Reporter vector (Promega, GA2550) was used as described in detail previously.^41^ Briefly, this vector encodes a fusion protein comprising human LC3B, a small N-terminal 11 amino acid HiBiT tag, and an intervening spacer segment that enhances the reporter’s specificity for the autophagic pathway. The reporter vector was transfected using Lipofectamine 2000 (Invitrogen), followed by selection with Geneticin (Gibco) at 800 µg ml^−^^1^. Stable LC3 HiBiT reporter cell lines were maintained at 400 µg ml^−^^1^ Geneticin and were seeded on 96-well plates for analysis. On the subsequent day, increasing concentrations of DCA or Metformin were administered, and LC3 HiBiT reporter activity was measured after 6 h using the Nano-Glo HiBiT Lytic Detection System (Promega, N3040) and an Orion II luminometer (Berthold), in accordance with the manufacturer’s instructions. A reduction in LC3 HiBiT reporter activity corresponds to autophagic LC3 degradation, thereby indicating autophagy induction. Alternatively, Chloroquine (50 µM) was used in separate experiments to hinder autophagosome-lysosome fusion, preventing LC3 HiBiT reporter degradation and resulting in reporter accumulation thereby measuring autophagic flux. To assess the influence of endogenous mTOR activity on drug-induced autophagy, LC3 HiBiT reporter assays were performed on cells treated with AZD8055 (0.5 µM). Both Chloroquine and AZD8055 treatments were initiated 48 h ahead of the 6-h exposure to metabolic drugs. Following background correction, luminescence signals were normalized to untreated samples.

### Luciferase assays with cell culture media and cell lysates

The lentiviral vectors for Gaussia and Cypridina Luciferase (GLuc and CLuc) and the retroviral vector containing Firefly-T2A-Gaussia Luciferases (FLuc-T2A-GLuc) were described previously.^44,45^ Briefly, the GLuc and CLuc lentiviral vectors were co-transfected with packaging plasmids (pMD2.G Addgene plasmid #12259 and psPAX2 Addgene plasmid #12260) into 293T cells using the calcium-phosphate technique. The retroviral FLuc-T2A-GLuc vector was transfected into Platinum-E cells (Cell Biolabs) utilizing a standard calcium-phosphate protocol. For infecting target cells, the supernatants containing lentiviral or retroviral particles were collected on the second and third-day post-transfection, filtered through a 0.45 μm filter, and then supplemented with 8 μg mL^−1^ polybrene for efficient infection. Cells that underwent transduction with GLuc or CLuc were subsequently selected with puromycin (2 μg ml^−1^) for 5 days and were maintained in 1 μg ml^−1^ puromycin. Cells that underwent transduction with FLuc-T2A-GLuc were selected with blasticidin (10 μg ml^−1^) for a period of 10 days and were maintained in this concentration.

Cells labeled with secreted luciferases (GLuc and CLuc) were seeded as a 1:1 mixture in triplicates on 24-well plates. Supernatants were collected every 24 h and substituted with fresh medium. These supernatants were stored in a 96-well plate at −20 °C. At the end of the experiment, all stored supernatants were thawed and subjected to shaking on a Thermomixer (Eppendorf) for 5 min at room temperature. After centrifugation, these supernatants were further diluted to 1:10–1:20 for luciferase activity measurements. A 10 mM stock of coelenterazine (PJK, Germany), the substrate for GLuc, was prepared in acidified ethanol (10 ml EtOH + 200 μl 6 M HCl). For CLuc, the substrate vargulin (NEB) was prepared following the manufacturer’s instructions. Subsequently, 5 μl of each appropriately diluted supernatant was measured in triplicates using white polypropylene 96-well plates with V-bottom (Greiner), using the Orion II luminometer (Berthold) with automated substrate injection. Either 50 μl coelenterazine solution (stock diluted 1:500 in phosphate-buffered saline (PBS)) or 25 μl vargulin solution (stock diluted 1:500 in Biolux Cypridina Luciferase Assay Buffer (NEB) prediluted 1:5 in PBS) was injected for the luminescence measurements. Intracellular firefly luciferase activity was quantified in cell lysates using the Beetle-Juice Firefly Luciferase assay (PJK) and Orion II luminometer (Berthold), following the manufacturer’s protocol.

### CRISPR-Cas9

For generating knockout clones two distinct single guide RNAs (sgRNAs) targeting *FIP200*, *ATG7*, *ATG14*, and *RUBCN* were designed and cloned into the plentiCRISPRv2 vector (Addgene #52961) using Golden Gate Cloning as described previously.^26,85^ The following sgRNAs were used: FIP200sg#1 CACCGCTGGTTAGGCACTCCAACAG, FIP200sg#2 CACCGAGGAGAGAGCACCAGTT-CAG, ATG7sg#1 CACCGAGAAATAATGGCGGCAGCTA, ATG7sg#2 CACCG TGCCCCTTTTAGTAGTGCCT, ATG14sg#1 CACCGTGAAGGCCTTCTCAAAACCA, ATG14sg#2 CACCGAGCTTTACAGTCGAGCACAA, RUBCNsg#3 CACCGACGCATCGATCTGAGCCAGG and RUBCNsg#4 CACCGCTGAGCATAGCTGTGGAG. For infecting T47D and MCF-7 cells, the supernatants containing lentiviral particles were collected on the second and third day post-transfection, filtered through a 0.45 μm filter, and then supplemented with 8 μg mL^−1^ polybrene for efficient infection. Cells that underwent transduction were subsequently selected with puromycin (2 μg ml^−1^) for a period of 5 days and were maintained in 1 μg ml^−1^ puromycin. Cells were single-cell cloned and analyzed for successful knockout by western blotting.

### Western blotting

Cells were lysed in NP-40 Lysis Buffer (50 mM Tris-HCl, 150 mM NaCl, 5 mM EDTA, 2% NP-40, pH 8.0) supplemented with protease inhibitor (complete ULTRA tablets EASYpack, Roche) and phosphatase inhibitor (PhosSTOP, Roche). Protein concentration was determined by Bradford assay (Bio-Rad). Total protein (20-50 μg) was separated on NuPAGE SDS Gels (Life Technologies) and tank-blotted to PVDF membranes. Following blocking in TBST (5 mM Tris, 15 mM NaCl, 0.1% Tween 20, pH 7.5) with 5% nonfat dry milk, membranes were incubated with primary antibodies diluted in TBST/5% nonfat dry milk and incubated overnight at 4 °C. Antibodies: Gaussia Luciferase/GLuc^+^ (1:1000, #401P, Nanolight), Cypridina Luciferase/CLuc^+^ (1:100, #IT-000-013, Immune Technology), Firefly Luciferase/FLuc^+^ (1:500, #3848-500, BioVision), cleaved PARP (Asp214) (1:1000, #9541, Cell Signaling), phospho-ULK1 (Ser757) (1:1000, #14202, Cell Signaling), total ULK (R600) (1:1000, #4773, Cell Signaling), p62/SQSTM1 (1:1000, P0067, Sigma), LC3B (LC3-I/II) (1:1000, ab48394, Abcam), phospho-p70S6Kinase (Thr389) (1:1000, 108D2 #9234, Cell Signaling), total p70S6Kinase (H9) (1:500, sc-8418, Santa Cruz Biotechnology), phospho-4E-BP1 (Thr37/46) (1:1000, 236B4 #2855, Cell Signaling), total 4E-BP1 (R-113) (1:200, #sc-6936, Santa Cruz Biotechnology) phospho-AMPK (Thr172) (40H9) (1:1000, #2535, Cell Signaling), total AMPK (23A3) (1:1000, #2603, Cell Signaling), phospho-Acetyl-CoA Carboxylase (Ser79) (1:1000, #3661, Cell Signaling), total Acetyl-CoA Carboxylase (C83B10) (1:1000, #3676, Cell Signaling), ATG7 (D12B11) (1:1000, #8558, Cell Signaling), FIP200 (D10D11) (1:1000, #12436, Cell Signaling), ATG14 (1:1000, #5504, Cell Signaling), RUBCN (D9F7) (1:1000, #8465, Cell Signaling), β-Actin (AC-15) (1:10.000, ab6276, Abcam). Proteins were detected with secondary antibody (anti-mouse IgG-HRP, anti-rabbit IgG-HRP from GE Healthcare, 1:3,000) using WesternBright ECL Substrat Sirius kit (Biozym) and anti-Goat IgG-Alexa 488 (1:1000, #A32814, Thermofisher).

### Metabolic analysis

Oxygen consumption rates (OCR) and extracellular acidification rates (ECAR) were measured using a Seahorse XFe96 Analyzer with Wave software (version 2.6.4.24, Agilent). On day 1, 12,500 cells in 80 µL of growth medium were seeded per well in a Seahorse XFe96/XF Pro Cell Culture Microplate (Agilent) and incubated overnight at 37 °C in a non-CO_2_ incubator. On day 2, cells were washed twice with assay medium (Seahorse XF DMEM Medium, pH 7.4, supplemented with 10 mM XF glucose, 1 mM XF pyruvate, and 2 mM XF glutamine), then pre-incubated for 45–60 min prior to analysis. The XF Real-Time ATP Rate Assay (Agilent) was conducted using a custom protocol: cells were exposed to 4 mM Metformin (Sigma) for 6 h, followed by the addition of 150 µM Oligomycin for 18 min, and finally 50 µM Rotenone/Antimycin A for an additional 18 min.

For untargeted LC/MS metabolomics, cells were treated with 4 mM Metformin. For the 2-day time point, 500,000 cells (T47D^Par^, T47D^AR^, T47D^ATG7^) were plated in triplicate for untreated samples on 10 cm dishes, while 1 × 10^6^ cells were plated in triplicate for the Metformin-treated condition. For the 5-day time point, 250,000 cells per condition were plated in triplicate for untreated samples, and 2 × 10^6^ cells were plated in triplicate for the Metformin-treated condition. For sample preparation and extraction, cells were washed three times with pre-warmed PBS (37 °C), placed on dry ice, and lysed. Metabolites were extracted by scraping cells with 1 mL of pre-cooled (−20 °C) 80% methanol solution. The extracts were transferred into 1.5 mL Eppendorf tubes and centrifuged at 16,000 rpm for 5 min at 4 °C. The supernatants were aliquoted (350 µL) for subsequent analysis using both reverse phase (RP) and hydrophilic interaction liquid chromatography (HILIC) methods, as well as for preparing a pooled quality control sample (250 µL) which was processed identically to the other samples. All samples were dried overnight in a speed vacuum and reconstituted in the appropriate RP and HILIC eluents for their respective analyses.

The metabolomic analyses were conducted using a Dionex Ultimate 3000 chromatography system coupled with a Q Exactive Focus mass spectrometer, equipped with a heated electrospray ionization (HESI) source and operated via TraceFinder 4.1 software (Thermo Fisher Scientific, Dreieich, Germany). Each sample underwent analysis in both RP and HILIC, with data collected in both positive and negative polarization. QC samples were injected every five samples to allow correcting for instrument variations.

For RP, the system utilized an Acquity UPLC BEH C18 column measuring 1.7 µm in particle size and 2.1 × 100 mm in dimensions, along with a 2.1 × 5 mm guard column to extend the column’s lifespan. The RP eluent system consisted of water with 0.1% formic acid as mobile phase A and methanol with 0.1% formic acid as mobile phase B. Chromatographic separation was achieved using a gradient program starting with 10% B, increased linearly to 98% B over 9 min. This concentration was maintained until 11 min, followed by a return to 10% B from 11.1 to 15 min for re-equilibration. For HILIC analysis, an Acquity UPLC BEH Amide column of the same dimensions (1.7 µm, 2.1 × 100 mm) was used, also with a 2.1 × 5 mm guard column. The HILIC eluent system comprised mobile phase A, a solution of 10 mM ammonium formate with 0.1% formic acid, and mobile phase B, 10 mM ammonium formate with 5% water, 95% acetonitrile, and 0.1% formic acid. The HILIC gradient program started with 100% B for the first 2 min, gradually decreasing to 30% B by the 14th min, and then reverting to the starting conditions by 16.5 min. For both chromatographic methods, the column temperature was consistently maintained at 40 °C, the flow rate was set at 0.35 mL/min and the injection volume was 2 µL.

The Q Exactive Focus mass spectrometer was operated with a capillary voltage of 3.5 kV in positive mode and −3 kV in negative mode. The capillary temperature was set to 380 °C, while the auxiliary gas temperature was 400 °C. The sheath gas pressure, auxiliary gas pressure, and sweep gas flow rate were set to 60, 20, and 0 arbitrary units, respectively. Nitrogen 5.0 was used for these gases. The scanning range was set from 66.7 to 1000 m/z. For full scan (MS1) analysis, the mass resolution was set to 70,000, with the automatic gain control (AGC) target set at 1e6 and the maximum injection time set to auto mode. In data-dependent MS2 (ddMS2) mode, the resolution was reduced to 35,000, with an AGC target of 5e4 and the maximum injection time also set to auto.

Data were analyzed using Compound Discoverer 3.3 (Thermo Fisher Scientific, Dreieich, Germany). Retention time tolerance for alignment and mass tolerance for annotation was set to 0.2 min and 5 ppm respectively. Compound annotations were made using mzcloud^TM^, mzVault, and ChemSpider databases. After pre-processing, peaks were manually filtered by re-checking individual chromatograms and spectral matching with reference libraries. Peak areas were normalized according to cell count and underwent further QC correction. The final peak list, merging results from HILIC and RP analyses in both positive and negative modes, was generated by selecting the entry with the highest abundance for compounds detected in multiple modes (Supplementary Table 1). This compound list was used for bioinformatic analysis using Python and MetaboAnalyst 6.0.^93^ The metabolomics raw data were submitted to the MetaboLights archive (www.ebi.ac.uk/metabolights)^94^ with accession number MTBLS11671.

### Targeted GC-MS analysis for L-aspartate

#### Sample preparation cell lysates

Cell lysates were prepared identically for untargeted metabolomics up to the drying step, with the only difference being the addition of an internal standard (1 nM ^13^C_4_-^15^N-aspartate). In short, cells were lysed using 80% MeOH, 10 µL of an internal standard added, the extracts centrifuged, supernatants transferred to another vial and dried in a speed vac at 4 °C overnight.

#### Sample preparation FFPE tumor samples

The punched pieces of the FFPE blocks were placed into 2 mL screw-cap vials. Subsequently, 1 mL of 80% MeOH was added, along with 10 µL of an internal standard (1 nM ^13^C_4_-^15^N-aspartate). Steel beads were introduced, and the mixture was homogenized using a Bead Blaster (Benchmark). After homogenization, the samples were centrifuged, and the supernatant was transferred to 1.5 mL Eppendorf tubes, followed by drying in a speed vac at 4 °C overnight.

#### Derivatization

Dried cell lysate extracts or FFPE extracts underwent derivatization for subsequent GC-MS analysis. For this, 50 µL of dichloromethane was added, and the samples were dried again at 20 °C. Afterward, 20 µL of 2% MOX (20 mg/mL methoxamine in pyridine) were added, and the samples were incubated in a thermomixer (Eppendorf) at 40 °C for 1.5 h. Finally, 20 µL of N-tert-butyldimethylsilyl-N-methyltrifluoroacetamide with 1% tert-butyldimethylchlorosilane (Merck AG Darmstadt) were added, and the mixtures were incubated again in the thermomixer at 50 °C for 3 h.

#### GC-MS analysis

All samples were analyzed using an Agilent 7890A gas chromatograph coupled to a 5975 mass spectrometer (Agilent, USA) equipped with an HP-5MS column (30 m × 250 μm × 0.25 μm; J&W Scientific, USA). The inlet was maintained at 250 °C and 10 psi with a septum purge flow of 3 mL/min and a 1:1 split ratio. Helium served as the carrier gas at a flow rate of 1 mL/min. A 1 µL injection volume was used, and the temperature program started with an oven temperature of 100 °C held for 1 min, followed by a ramp of 12.5 °C/min to 300 °C, which was held for 1.5 min. The electron energy was set to 70 eV, with an electron current of 34.6 µA. The MS source and quadrupole temperatures were set to 230 °C and 150 °C, respectively. Mass spectra were acquired in selected ion monitoring (SIM) mode for asp (m/z 418, 390, 316) and ^13^C_4_-^15^N-asp (m/z 423, 394). Data analysis was conducted using Mass Hunter Quantitative Analysis software (version B.05.00).

### Animals

All xenograft experiments were performed according to the German animal welfare law and the European legislation for the protection of animals used for scientific purposes (2010/63/EU) and were approved by the regional board (RP Giessen). Animal group sizes were calculated by an a priori power analysis. All experiments were done in 14–34 week-old, female *NOD.Cg-Prkdc*^*scid*^ *Il2rg*^*tm1Wjl*^ *Tg(CMV-IL3,CSF2,KITLG)1Eav/MloySzJ* mice. Mice were kept under specified pathogen-free (SPF) conditions in individually ventilated cages as groups with a 12–12 h light–dark cycle and a standard Altromin housing diet ad libitum. For monitoring the in vivo growth of parental T47D^Par^, Alpelisib-resistant (T47D^AR1^), and autophagy-deficient T47D^ATG7^ cells in a competitive setting, T47D^Par^ cells were labeled ex vivo with CLuc lentivirus and T47D^AR1^ and T47D^ATG7^ cells with GLuc-FLuc retrovirus as previously described. CLuc and GLuc luciferases are secreted by tumor cells and their activity in blood samples quantitatively reflects the total amount of viable tumor cells in the mouse.^44,95^

One week before tumor cell injection, a 17β-Estradiol pellet (Belma technologies) was implanted subcutaneously. The orthotopic injection of breast cancer cells into the mammary fat pad was described in detail in previous studies.^65,96^ Briefly, mice were anesthetized using Isoflurane (Baxter), and the abdominal area around the inguinal mammary fat pad was disinfected with octenisept (Schülke). The 1:1 cell suspension (T47D^Par^:T47D^AR1^ cells or T47D^Par^:T47D^ATG7^ cells) was orthotopically injected in the mammary fat pad (in total 1 ×10^6^ cells) with 50 μL growth factor-reduced Matrigel (Corning).

Two weeks after tumor cell injection, mice were randomly allocated to the treatment cohorts, and Metformin (75 mg kg^−^^1^ body weight), DCA (250 mg kg^−^^1^ body weight), Metformin, and DCA (75 mg kg^−^^1^ and 250 mg kg^−^^1^ body weight) and Alpelisib (35 mg kg^−^^1^ body weight) were administered orally once a day. Control mice received H_2_O as vehicle control. For monitoring of tumor growth, 20 µl of blood was obtained by tail vein puncture and mixed directly with 4 µl of 0.125 IE ml^−^^1^ heparin. Plasma was collected by centrifugation (15 min, 3600 g, 4 °C). All collected plasma samples were stored at −20 °C and measured together at the end of the experiment with a single batch of reagents. For luciferase activity measurements in the Orion II luminometer (Berthold), plasma was diluted 1:10–1:20 with PBS. Each diluted sample (5 µl) was measured by injection of 100 µl coelenterazine (stock from PJK, Germany, diluted 1:200 dilution in PBS) or 25 µl vargulin reagent (stock from NEB diluted 1:200 in Biolux Cypridina Luciferase Assay Buffer prediluted 1:5 in PBS).

For analyzing short-term responses to multiple metabolic drugs and Alpelisib, mice were injected with T47D^Par^, T47D^AR1^, or T47D^ATG7^ cells orthotopically in the mammary fat pad (in total 1 × 10^6^ cells) with 50 μL growth factor-reduced Matrigel (Corning).

After 2 weeks of tumor growth, animals were treated for 3 days with the above-mentioned drugs in the same dosages (*n* = 3 per cohort). 3 days after the start of treatment, animals were sacrificed and their mammary tumors were histologically analyzed.

### Luciferase assays with tumor lysates

Breast tumors were excised from euthanized mice and minced. 10–20 mg of the breast tumor was lysed in 100 μl passive lysis buffer (Promega) with the TissueLyser LT (Qiagen). Tumor lysate (5 μl) was measured in duplicate measurements for GLuc, CLuc, and FLuc activity without background correction as described for blood plasma.

### Bioluminescence imaging

D-Luciferin (Abcam) was dissolved in sterile PBS (15 mg/mL), and 200 µL was injected intraperitoneally. Mice were anesthetized with Isoflurane (Baxter). After a waiting time of 5 min, mice were imaged for 60 s using an In-Vivo Xtreme II imager (Bruker).

### Immunohistochemistry

For immunohistochemistry, we performed heat-induced epitope retrieval with citrate (for p62 and phospho-4E-BP1), EDTA (for cleaved Caspase-3 and phospho-ACC) or Trilogy (for phospho-Histone H3, Ki67, and phospho-AKT). Staining was performed on a Dako Autostainer Plus. Sections were incubated for 45 min with the following primary antibodies: rabbit anti-Cleaved Caspase-3 Asp175 (1:200; Cell Signaling), rabbit anti-Phospho-Acetyl-CoA-Carboxylase Ser79 (1:500; Cell Signaling), rabbit anti-Phospho-Histone H3 Ser10 (1:200; Cell Signaling), rabbit anti-Ki67 (1:75; Abcam ab15580), rabbit anti-Phospho-AKT Ser473 (1:25; Cell Signaling), mouse anti-p62 (1:20,000; Abcam ab56416) and rabbit anti-LC3B-II (1:2000; Abcam ab48394). Sections were washed and incubated with Dako REAL EnVision Detection System according to the manufacturer´s protocol and counterstained with hematoxylin.

### Immunohistochemical staining and evaluation with VM Slide Explorer 2.2

Accumulation of p62 and phosphorylation of 4E-BP1^T37/46^ on the tumor was evaluated on a tissue microarray (TMA) using therapy-naïve archived tumor tissue from the adjuvant, multicenter, German Breast Group (GBG) GAIN-trial.^67^ Immunohistochemical staining was performed at the Institute of Pathology, Philipps-University Marburg, UKGM University Hospital Marburg on a DAKO Autostainer Link 48. Here, p62 and p4E-BP1^T37/46^ levels could be assessed on *n* = 1120 eligible TMA cores with corresponding clinical outcome information of *n* = 687 HR^+^/Her2^−^ BC patients (Supplementary Fig. 6). The research project was approved by the Ethics Committee of the University of Marburg (Ethics Opinion No 38/20). Deparaffinization of the TMA-slides was followed by antigen treatment with Target Retrieval Solution Citrate (pH 6.0, S 2369 Fa. Dako) for 30 min in a pressure cooker. After blocking with Peroxidase-Blocking Solution (S2023; Dako), incubation with 1:1000 diluted anti-p62 antibody (mouse mAB; clone 2C11; Abcam) and 1:200 diluted anti-p4E-BP1^T37/46^ antibody (rabbit mAb; clone 236B4, Cell Signaling) was performed. DAKO REAL EnVision HRP Rabbit/Mouse (K5007; Dako) was used as the secondary antibody. The chromogen (K5007; Dako) reacted with HRP, resulting in a color change. The slides were digitized with an Aperio AT2 Slide Scanner (Leica Biosystems). Cytoplasmic levels of p62 and p4E-BP1^T37/46^ were evaluated and calculated by IRS score (intensity of staining x percentage of positive cells) using VM Slide Explorer 2.2. Cutoff values for binary classification (p4E-BP1^T37/46^ and p62 low and high) were determined by “Cutoff Finder” (https://molpathoheidelberg.shinyapps.io/CutoffFinder_v1/).

### Clinical data reporting and statistical analysis

Statistical analyses for biomarker analyses were performed with SPSS statistics, version 29.0.0.9 (241, IBM Corp, Armonk, NY, USA). *P*-values of hypothesis tests of associations were determined in a 2 × 2 matrix using Fisher’s exact test (two-sided) and for matrices >2 × 2 using the Chi-square test. To stratify patients into groups of low and high biomarker expression, the optimal cutoff value with regard to OS was set using the Cutoff Finder web application (https://molpathoheidelberg.shinyapps.io/CutoffFinder_v1/). Cutoff Finder analysis delivered an IRS score of 5 as the optimal cutoff for p4E-BP1^T37/46^ and p62 on the tumor. Log-rank test was applied to calculate p-values and consequently to illustrate differences in survival and to examine the prognostic effect of biomarker expression. *p*-values < 0.05 were considered statistically significant.

For the animal experiments, an a priori power analysis was conducted to determine the necessary group sizes to detect an estimated effect size (Cohen’s d) of 1.5 for the first trial (Fig. 5) and 1.0 for the second trial (Fig. 6) with adequate statistical power (α = 0.05, 1−β = 0.80). The experiments were not randomized, and investigators were not blinded to allocation during the experiments and outcome assessment. For in vitro experiments, no statistical methods were used to predetermine sample size. GraphPad Prism software (version 10.2.3) was used to generate all plots and perform statistical analyses. p62 and LC3B-I/LC3B-II levels were quantified from Western blots using ImageJ software (version 1.51). Graphics were assembled using Adobe Illustrator (version 28.5) and BioRender.com (Figs. 1a, 6a, 7a and Supplementary Fig. 6).

The results presented in the graphs represent the mean or median values obtained from n replicates. Error bars in the figures indicate the standard deviation (SD) unless stated otherwise. Experiments investigating the interaction of two variables (e.g. treatment) were analyzed using two-way analysis of variance (ANOVA) followed by multiple comparison testing according to Tukey or Dunnett, as indicated. The ANOVA results and selected pairwise comparisons are reported in the figures. A *p*-value of less than 0.05 was considered statistically significant. Time course experiments were analyzed using multiple two-sided *t*-tests in combination with the false discovery rate approach (two-stage linear step-up procedure of Benjamini, Krieger, and Yekutieli). FDR *q* values < 0.05 are considered significant and are reported for the last time point or highest dose. When results from representative experiments are shown (e.g., Western blots, clonogenic growth assays, and luciferase assays), these were replicated in at least two independent experiments with similar results. Additionally, all experiments with T47D^AR^ cells were reproduced with an independently generated Alpelisib-resistant T47D cell clone and yielded similar results.

## Supplementary information

Supplementary Materials

Dataset 1

## Data Availability

All data generated or analyzed during this study are included in this published article (and its supplementary information file). The following source western blot data are provided with this paper: uncropped images for Figs. 2a, 3a, 4a, 4f, 6f, 7f, S3a, and S5a. The metabolomics data generated and analyzed for this paper have been archived in the MetaboLights database (www.ebi.ac.uk/metabolights) and are accessible under the accession code MTBLS11671.
