# Supplementary material for: Targeting PI3K inhibitor resistance in breast cancer with metabolic drugs
Source: Signal Transduct Target Ther. 2025 Mar 21;10:92. doi: 10.1038/s41392-025-02180-4 (PMC11926384; doi:10.1038/s41392-025-02180-4)
Supplement: Supplementary file 1 — Supplementary Materials [file 41392_2025_2180_MOESM1_ESM.docx]

Supplementary Materials for

**Targeting PI3K inhibitor resistance in breast cancer with metabolic drugs**

Niklas Gremke, Isabelle Besong, Alina Stroh, Luise von Wichert, Marie Witt, Sabrina Elmshäuser, Michael Wanzel, Martin F. Fromm, R. Verena Taudte, Sabine Schmatloch, Thomas Karn, Mattea Reinisch, Nader Hirmas, Sibylle Loibl, Thomas Wündisch, Anne-Sophie Litmeyer, Paul Jank, Carsten Denkert, Sebastian Griewing, Uwe Wagner and Thorsten Stiewe

Correspondence to: [Gremken@staff.uni-marburg.de](mailto:Gremken@staff.uni-marburg.de)

**This PDF file includes:**

Figures. S1 to S9

Captions for Figures S1 to S9

Table S1 (separate file)


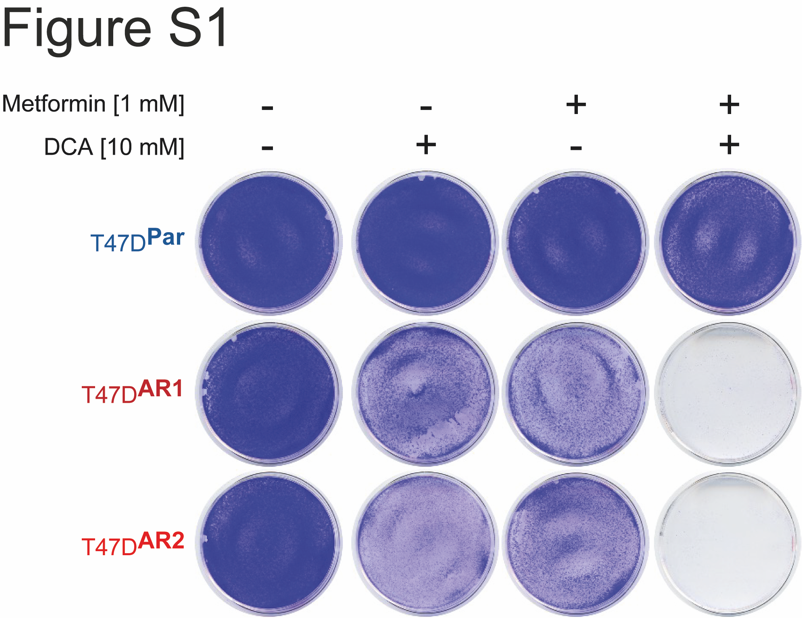


Figure. S1.

Potentiating effects of Metformin and DCA in Alpelisib-resistant breast cancer cells. Clonogenic growth of parental T47D^Par^ and Alpelisib-resistant T47D^AR1^ and T47D^AR2^ cells treated with Metformin and DCA in indicated concentrations.


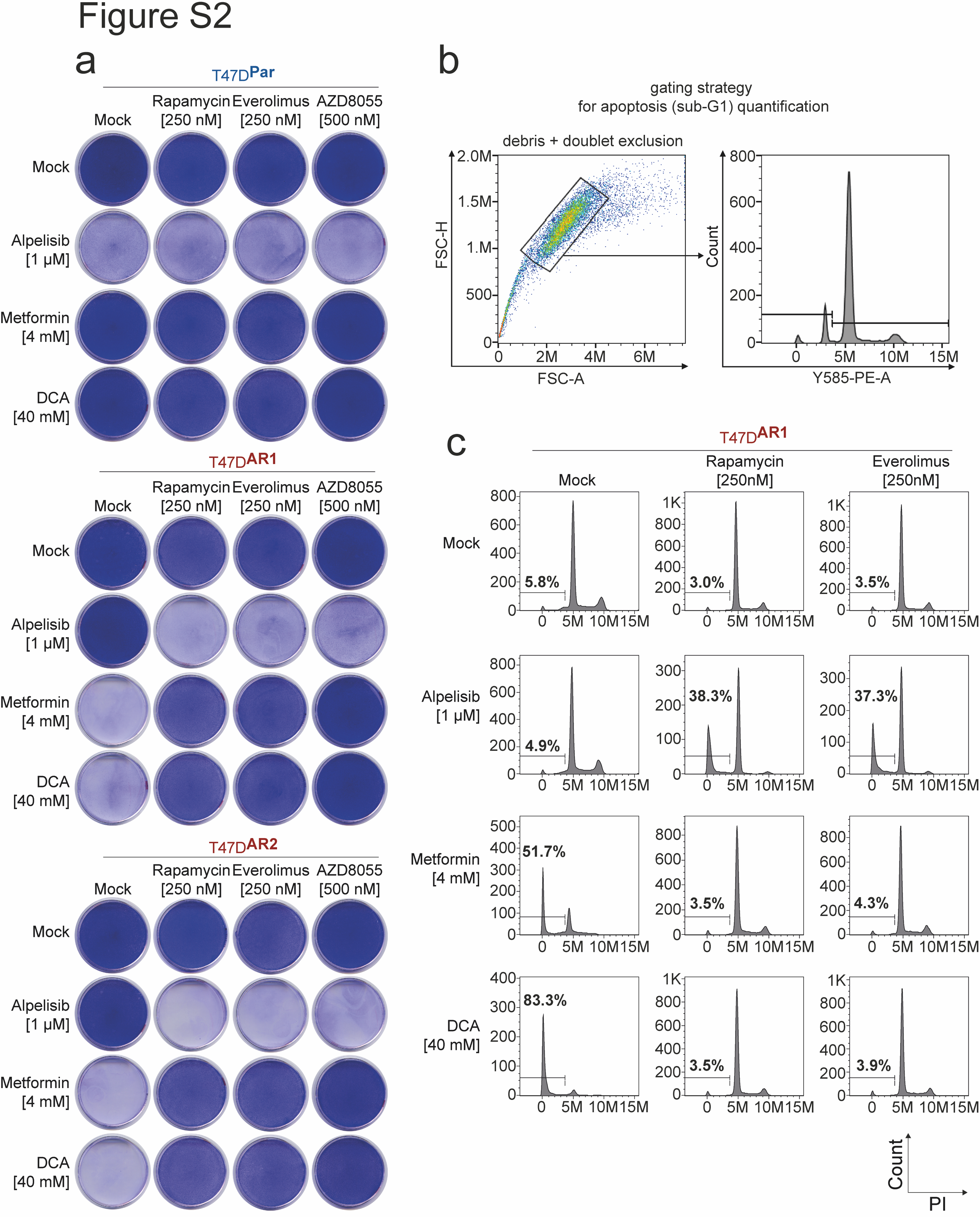


Figure. S2.

mTORC1 inhibitors resensitize T47D^AR1^ and T47D^AR2^ cells to Alpelisib but counteract the effect of DCA and Metformin. (a) Clonogenic growth assay of parental and Alpelisib-resistant T47D^AR1^ and T47D^AR2^ cells treated with DCA, Metformin, mTORC1 inhibitors (Rapamycin, Everolimus), and the dual mTOR inhibitor AZD8055 at the indicated concentrations and combinations. (b) Gating strategy. (c) Apoptosis assay. Shown are propidium iodide (PI) staining profiles with the apoptotic sub-G1 fraction of T47D^AR1^ cells indicated in %.


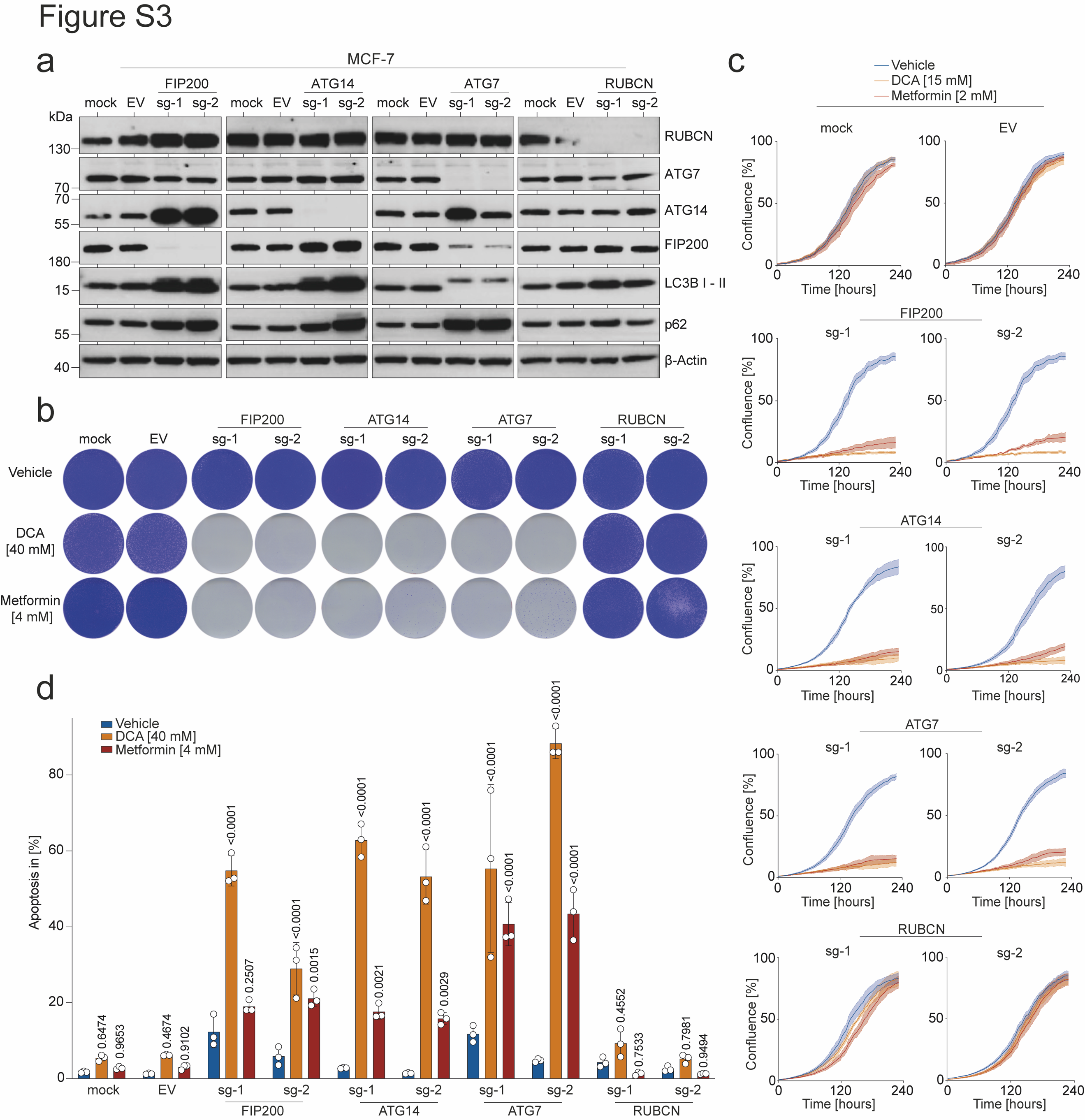


Figure. S3.

Metabolic vulnerability of CRISPR/Cas9-engineered autophagy-deficient MCF-7 breast cancer cells. (a – d) MCF-7 cells were infected with plentiCRISPRv2 vectors targeting *FIP-200*, *ATG7*, *ATG14* and *RUBCN* (each with two independent sgRNAs). After lentiviral transduction and puromycin selection, cells were single-cell cloned and examined for target gene knockout by Western blot (a). Mock: non-infected cells; EV: Empty vector control. (b) Clonogenic growth of control (mock and EV) and knockout cells treated with DCA or Metformin. (c) Real-time live cell imaging of control (mock and EV) and knockout cells treated with DCA or Metformin. Shown is the mean confluence in % over time ± SD (*n* = 3). (d) Flow cytometry analysis for apoptosis (sub-G1). Indicated knockout cells were treated with DCA or Metformin for 5 days. Shown are mean ± SD, *n* = 3, two-way ANOVA with Tukey’s multiple comparisons test.


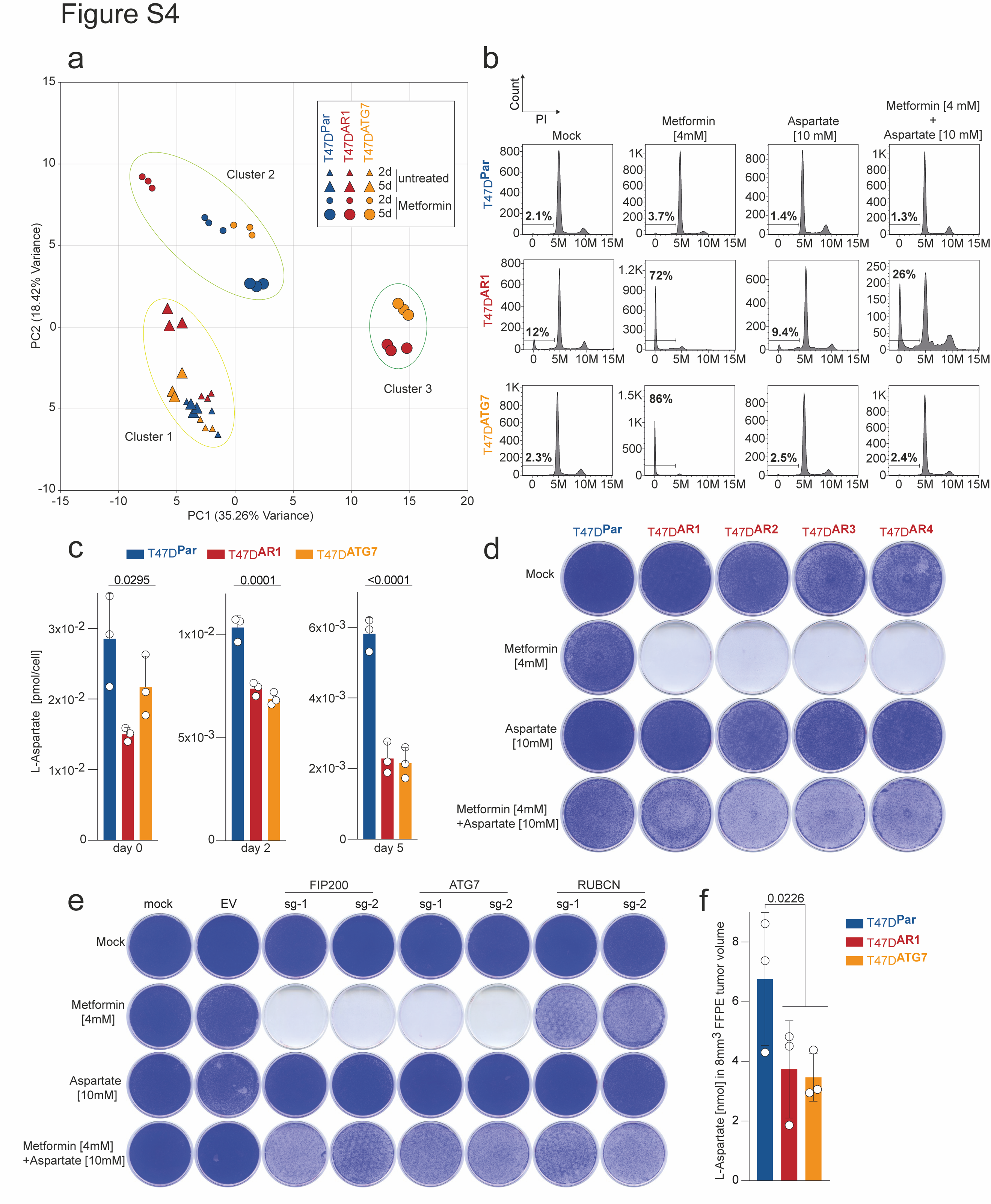


Figure S4.

Autophagy-deficiency exacerbates metabolic stress induced by metabolic drugs.

(**a**) Principal component (PC) analysis of metabolomic profiles from untreated and Metformin-treated T47D^Par^, T47D^AR1^, and T47D^ATG7^ cells at 2 and 5 days post-treatment. The scatter plot displays the first two PCs, with axis titles indicating the percentage of total variance explained by each component. Clusters 1-3 correspond to the hierarchical clustering results shown in Figure 5b. (**b**) Apoptosis rescue of Metformin vulnerability by supplementation with 10 mM L-aspartate. Shown are propidium iodide (PI) staining profiles with the apoptotic sub-G1 fraction indicated in %. (**c**) Gas chromatography–mass spectrometry (GC-MS) measurement of L-aspartate using samples of Figure 5e. Shown is L-aspartate in pmol per cell in the metformin-treated cells at day 0, 2 and 5. Data are presented as mean ± SD (n = 3). Statistical significance was determined by one-way ANOVA. (**d**) Clonogenic growth analysis of parental and Alpelisib-resistant cells demonstrates the rescue of Metformin-induced vulnerability upon supplementation with 10 mM L-aspartate. Representative images are presented. (**e**) Clonogenic growth of control cells (mock and EV) and T47D *FIP200*, *ATG7*, and *RUBCN* knockout cells, demonstrating the rescue of Metformin-induced vulnerability upon supplementation with 10 mM L-aspartate. Representative images are shown. (**f**) GC-MS measurement of L-aspartate using formalin-fixed paraffin-embedded (FFPE) tumors treated with metformin for 3 days from Figure 6 g – h and 7 g – h. Shown is L-aspartate in nmol per 8mm^3^ tumor volume. For the Metformin-treated cohort, n = 3 parental, Alpelisib-resistant, and ATG7-knockout tumors were available for each group. Statistical significance was determined by unpaired t test.


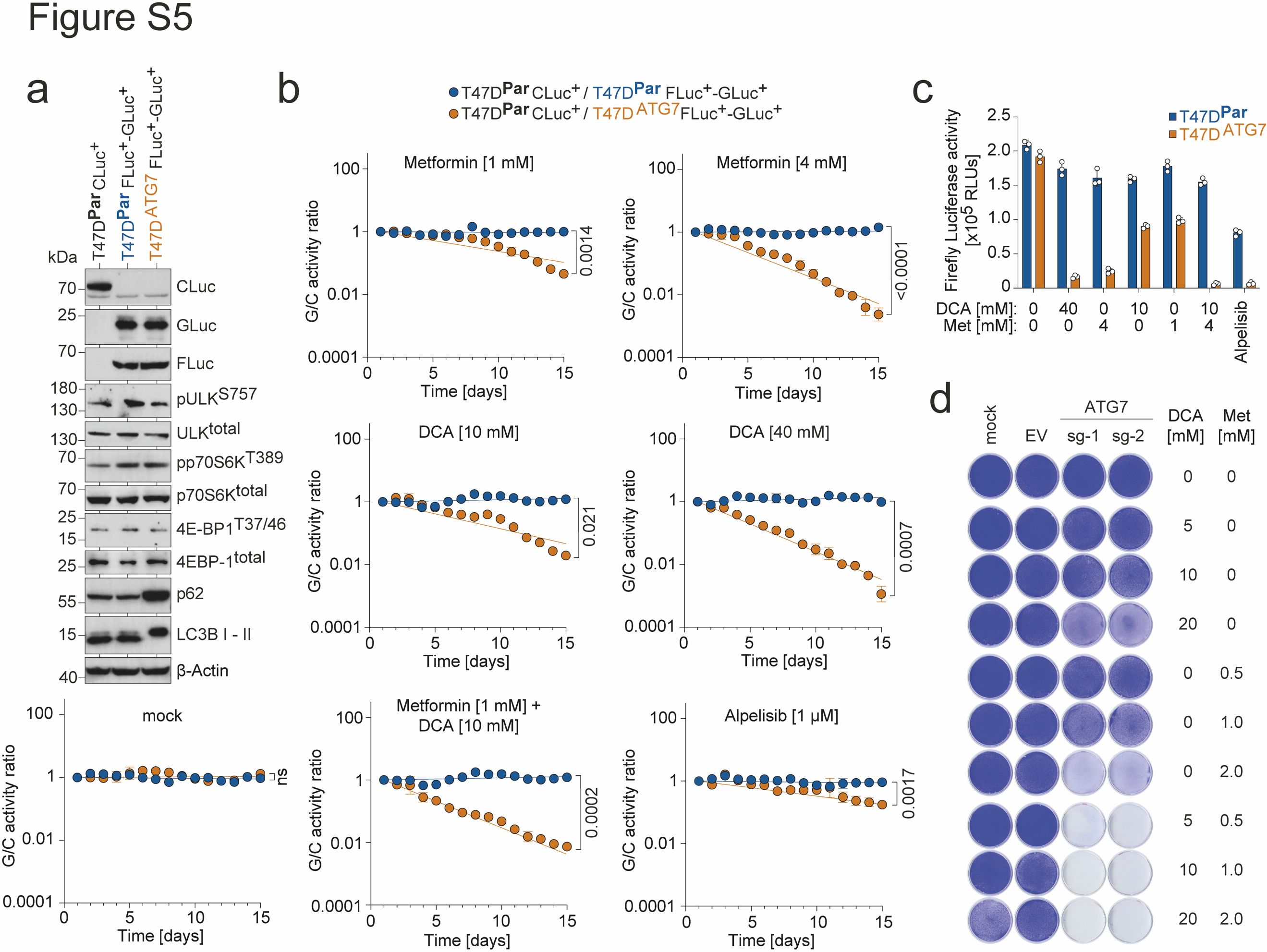


Figure S5.

*In vitro* proliferation competition assay of ATG7-knockout breast cancer cells. (a) Western blot of parental T47D^Par^ and autophagy deficient T47D^ATG7^ cells infected with indicated luciferase vectors. (b – c) T47D^Par^ cells were labelled with either CLuc (T47D^Par^ CLuc^+^) or FLuc-GLuc (T47D^Par^ FLuc^+^-GLuc^+^) and T47D^ATG7^ cells with FLuc-GLuc (T47D^ATG7^ FLuc^+^-GLuc^+^). For Control-Suspension T47D^Par^ CLuc^+^ and T47D^Par^ FLuc^+^-GLuc^+^ cells and for Test-Suspension T47D^Par^CLuc^+^ and T47D^ATG7^ FLuc^+^-GLuc^+^ cells were mixed in a 1:1 ratio, treated 15 days with indicated drugs and monitored daily for GLuc/CLuc activity (G/C activity) in the culture supernatant. (b) Shown is the G/C ratio normalized to day 1 ± SD, *n* = 3, FDR q values. (c) Endpoint measurement of intracellular FLuc activity within the Control- and Test-Suspension after 15 day treatment with indicated drugs. Shown are mean ± SD, *n* =3. (d) Clonogenic growth of control (mock and EV) and ATG7-knockout cells treated with DCA or Metformin at indicated concentrations. Mock: non-infected cells; EV: Empty vector control.


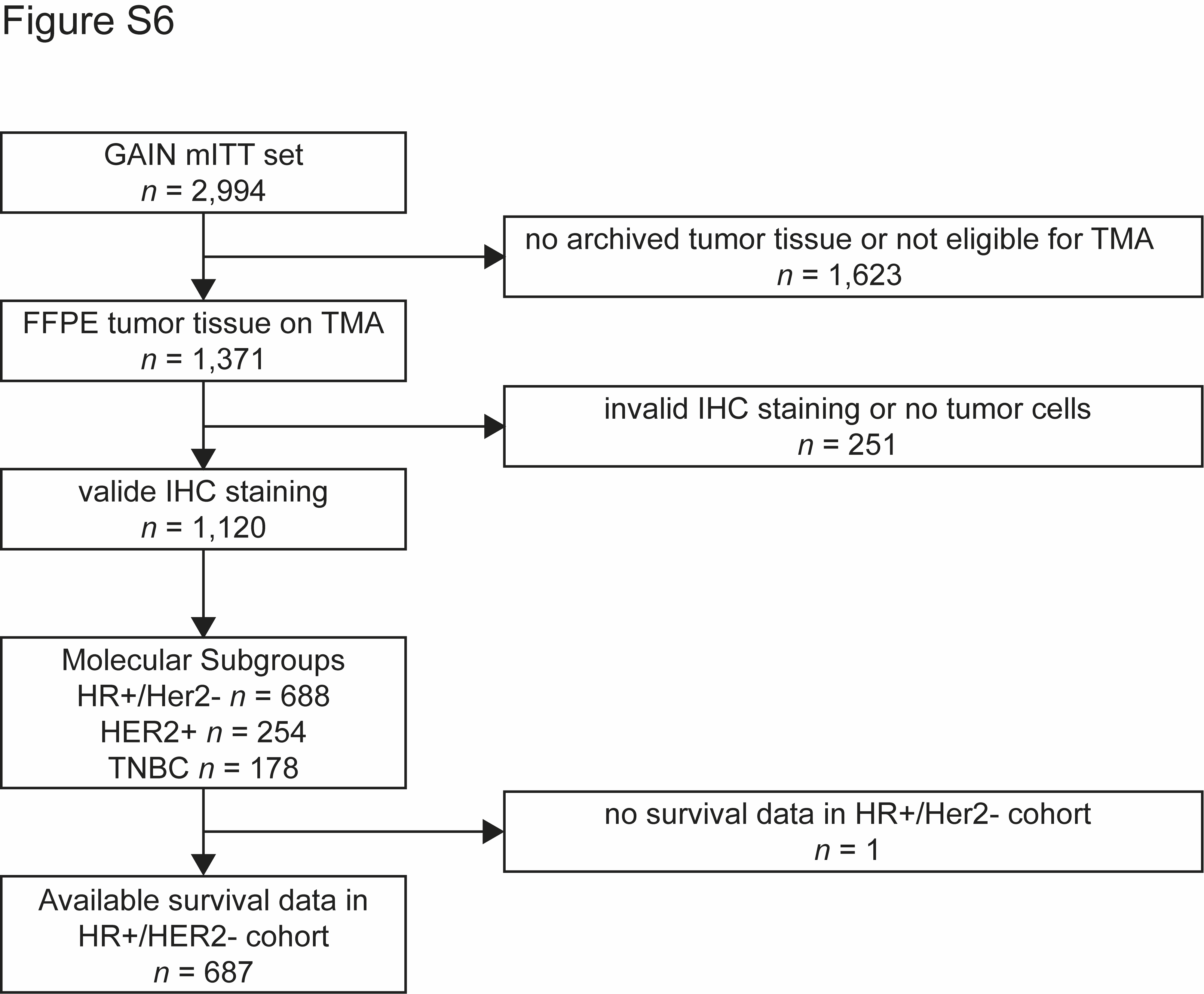


Figure S6.

CONSORT statement. Flow of samples from GAIN-trial specific modified intention to treat set (mITT) to eligible TMA samples (*n* = 1,120) and corresponding survival data of the HR^+^/Her2- cohort (*n* = 687).


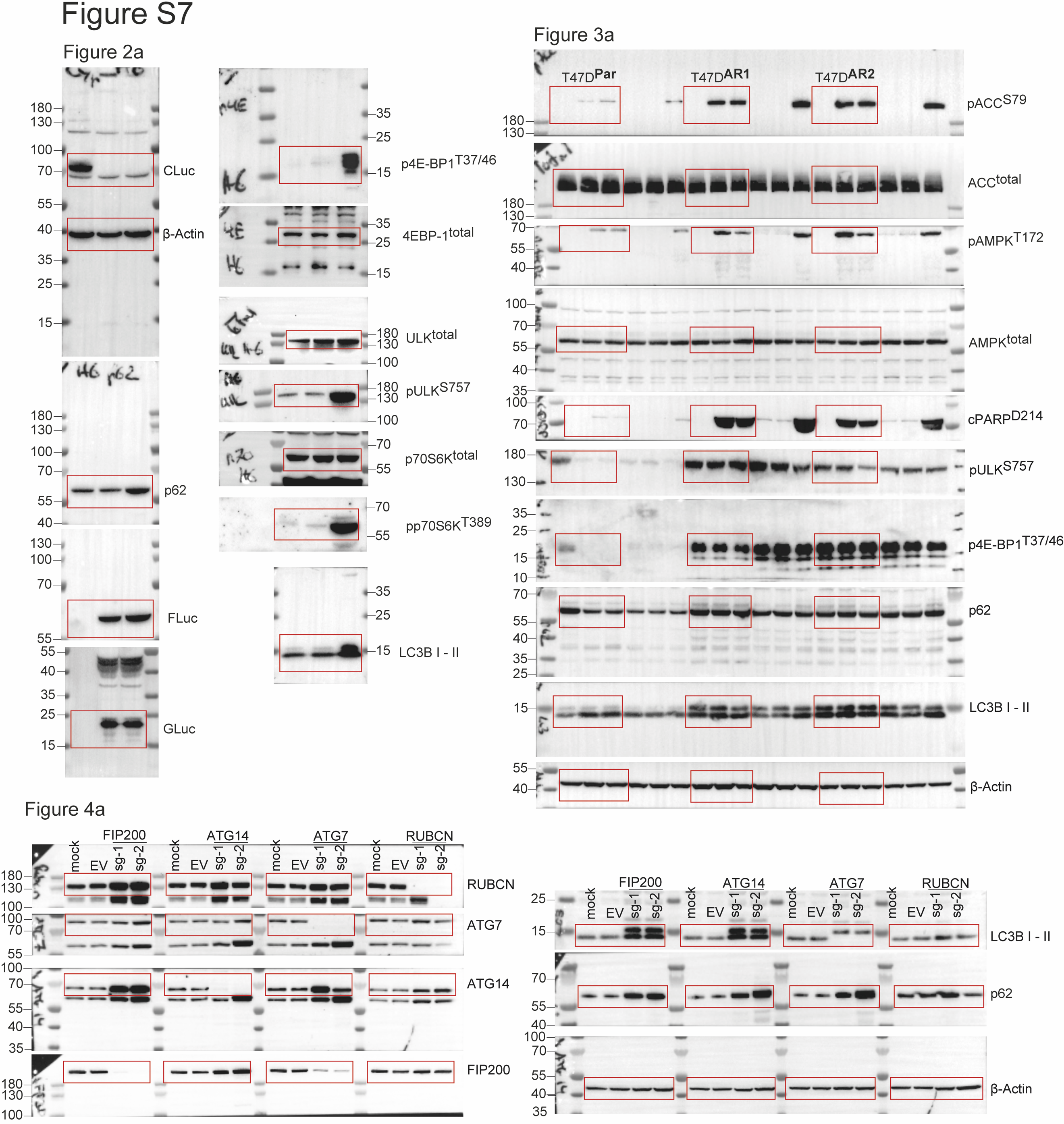


Figure S7.

Full western blot membranes used for figures 2a, 3a and 4a. The specific bands shown in the main figures are highlighted in red.


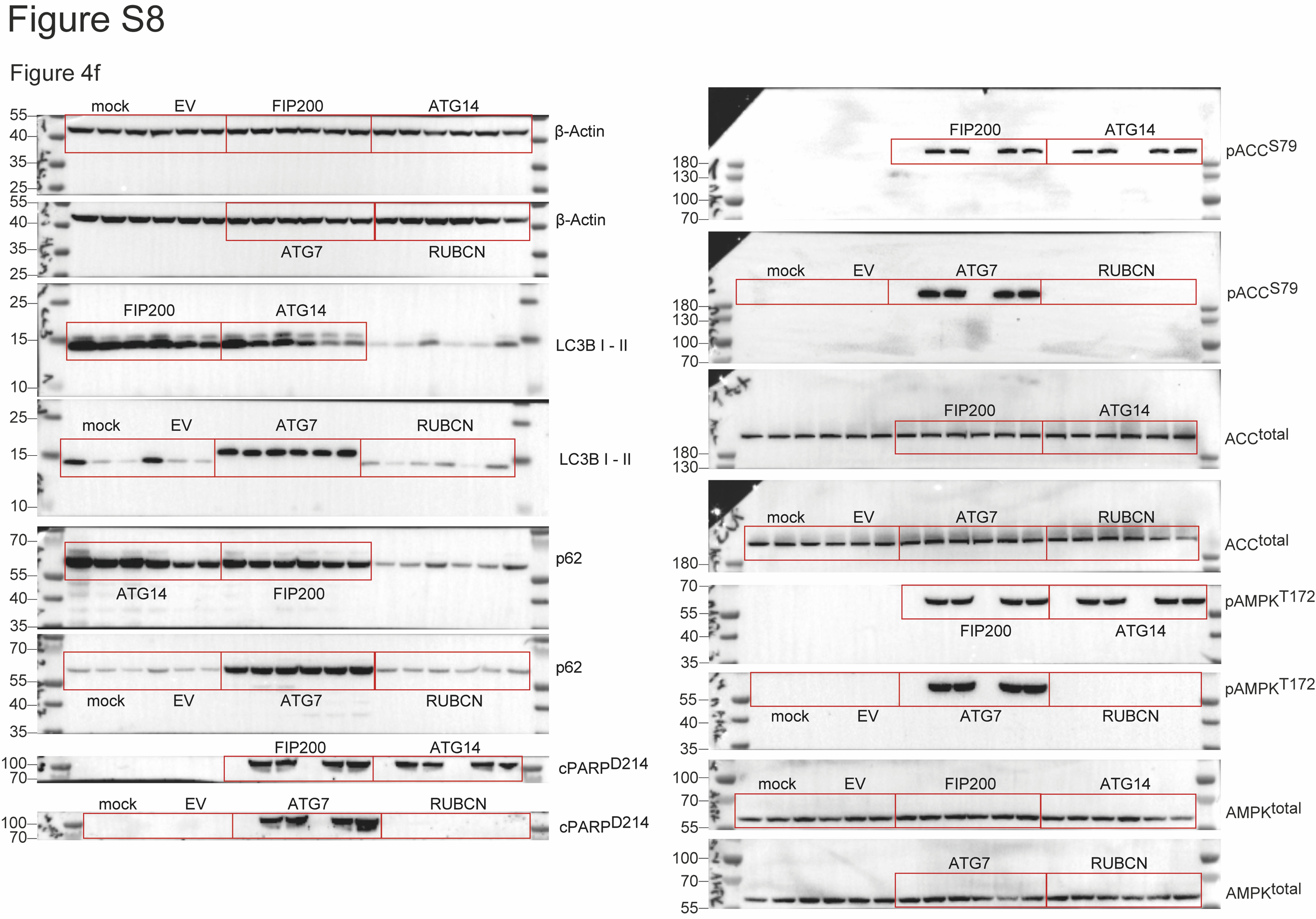


Figure S8.

Full western blot membranes used for figure 4f. The specific bands shown in the main figure are highlighted in red.


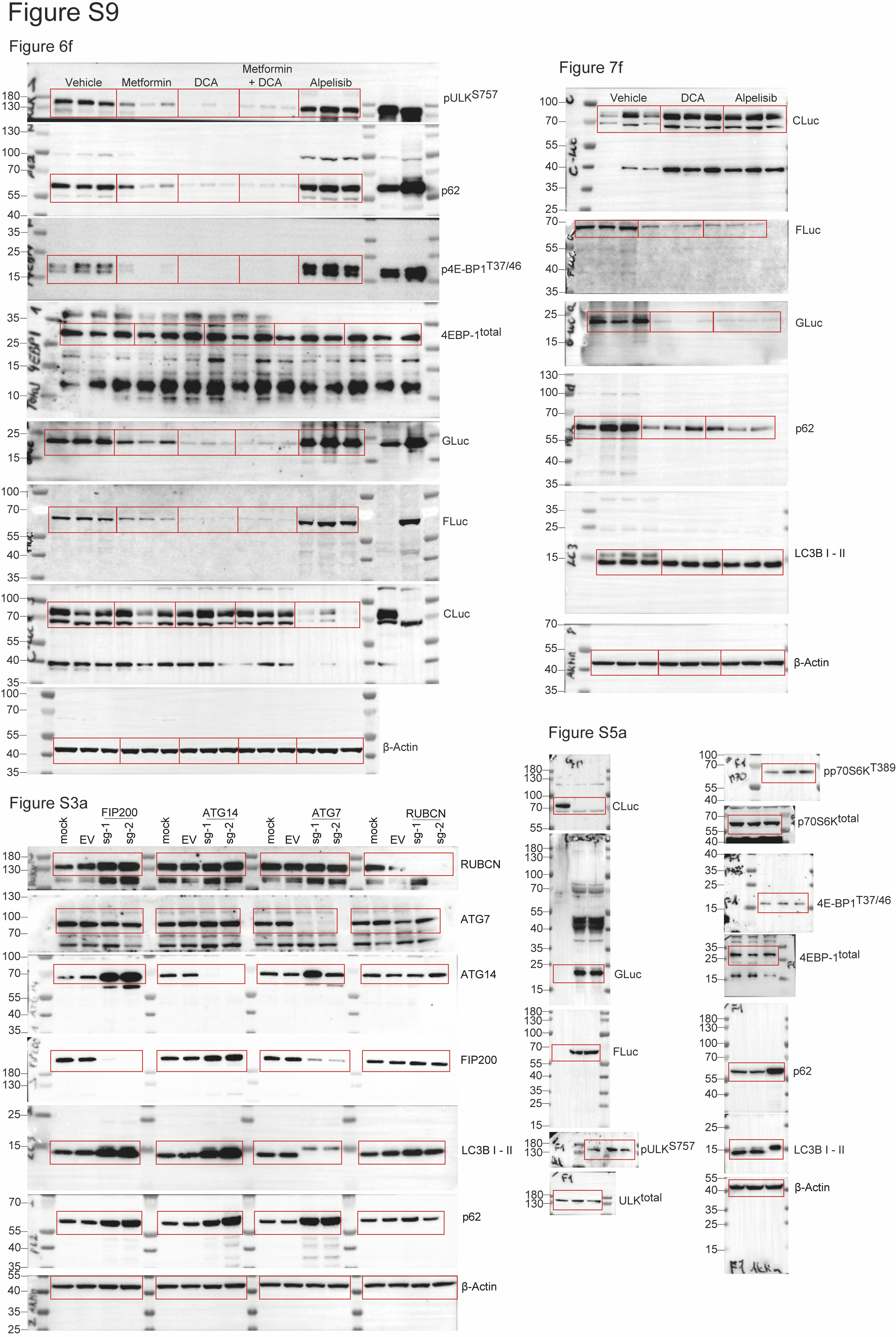


Figure S9.

Full western blot membranes used for figures 6f, 7f, S3a and S5a. The specific bands shown in the main figures are highlighted in red.

Table S1. (separate file)

Compound list used for bioinformatic analysis with Python and MetaboAnalyst 6.0.
